# Growth-Dependent Predation and Generalized Transduction of Antimicrobial Resistance by Bacteriophage

**DOI:** 10.1128/msystems.00135-22

**Published:** 2022-03-21

**Authors:** Quentin J. Leclerc, Jacob Wildfire, Arya Gupta, Jodi A. Lindsay, Gwenan M. Knight

**Affiliations:** a Centre for Mathematical Modelling of Infectious Diseases, Department of Infectious Disease Epidemiology, Faculty of Epidemiology & Population Health, London School of Hygiene & Tropical Medicine, London, United Kingdom; b Antimicrobial Resistance Centre, London School of Hygiene & Tropical Medicine, London, United Kingdom; c Institute for Infection & Immunity, St. George’s University of London, London, United Kingdom; University of California San Diego

**Keywords:** antimicrobial resistance, bacteriophages, horizontal gene transfer, mathematical modelling, microbiology, *Staphylococcus aureus*, transduction

## Abstract

Bacteriophage (phage) are both predators and evolutionary drivers for bacteria, notably contributing to the spread of antimicrobial resistance (AMR) genes by generalized transduction. Our current understanding of this complex relationship is limited. We used an interdisciplinary approach to quantify how these interacting dynamics can lead to the evolution of multidrug-resistant bacteria. We cocultured two strains of methicillin-resistant Staphylococcus aureus, each harboring a different antibiotic resistance gene, with generalized transducing phage. After a growth phase of 8 h, bacteria and phage surprisingly coexisted at a stable equilibrium in our culture, the level of which was dependent on the starting concentration of phage. We detected double-resistant bacteria as early as 7 h, indicating that transduction of AMR genes had occurred. We developed multiple mathematical models of the bacteria and phage relationship and found that phage-bacteria dynamics were best captured by a model in which phage burst size decreases as the bacteria population reaches stationary phase and where phage predation is frequency-dependent. We estimated that one in every 10^8^ new phage generated was a transducing phage carrying an AMR gene and that double-resistant bacteria were always predominantly generated by transduction rather than by growth. Our results suggest a shift in how we understand and model phage-bacteria dynamics. Although rates of generalized transduction could be interpreted as too rare to be significant, they are sufficient in our system to consistently lead to the evolution of multidrug-resistant bacteria. Currently, the potential of phage to contribute to the growing burden of AMR is likely underestimated.

**IMPORTANCE** Bacteriophage (phage), viruses that can infect and kill bacteria, are being investigated through phage therapy as a potential solution to the threat of antimicrobial resistance (AMR). In reality, however, phage are also natural drivers of bacterial evolution by transduction when they accidentally carry nonphage DNA between bacteria. Using laboratory work and mathematical models, we show that transduction leads to evolution of multidrug-resistant bacteria in less than 8 h and that phage production decreases when bacterial growth decreases, allowing bacteria and phage to coexist at stable equilibria. The joint dynamics of phage predation and transduction lead to complex interactions with bacteria, which must be clarified to prevent phage from contributing to the spread of AMR.

## INTRODUCTION

Bacteriophage (or phage) are major bacterial predators and the most abundant biological entities on the planet (1). However, phage are also natural drivers of bacterial evolution through horizontal gene transfer by transduction (2, 3). Antimicrobial resistance (AMR) genes can be transferred by transduction at high rates both *in vitro* and *in vivo* (4–6), meaning that phage may be substantially contributing to the rapidly increasing global public health threat of AMR (7). However, our understanding of these joint dynamics of predation and transduction and how to best represent them is limited.

**FIG 1:**
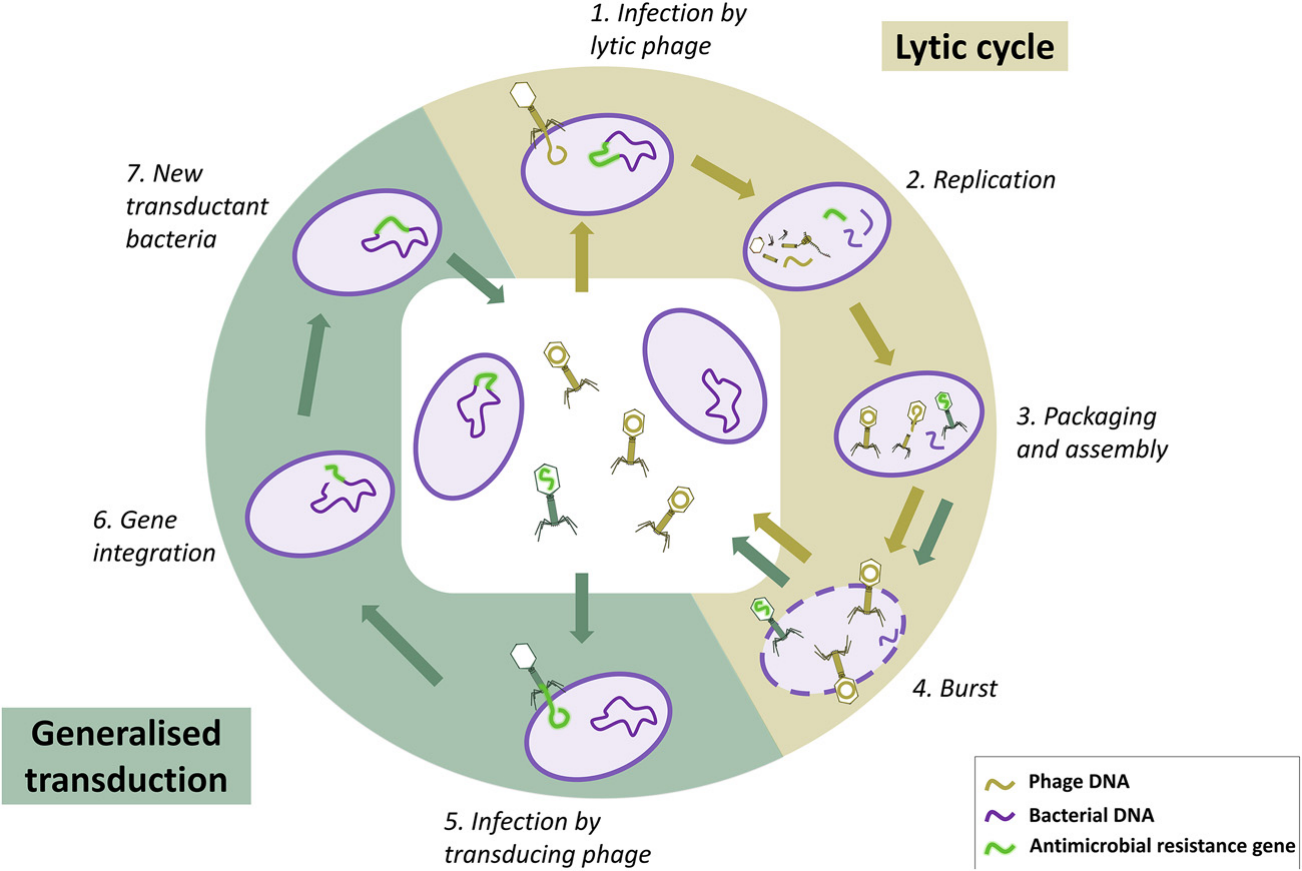
Phage lytic cycle and generalized transduction. In this environment, only some bacteria carry an antimicrobial resistance (AMR) gene (shown in green). The lytic cycle starts when a lytic phage infects a bacterium by binding and injecting its DNA (1). Phage molecules degrade bacterial DNA and utilize bacterial resources to create new phage components and replicate (2). These components are then assembled to form new phage particles (3). At this stage, bacterial DNA left in the cell can be packaged by mistake instead of phage DNA, which creates a transducing phage and starts the process of generalized transduction. In our example, the transducing phage carries the AMR gene. After a latent period of typically several minutes, the phage trigger lysis of the bacterium, bursting it and releasing the phage (4). The transducing phage can infect another bacterium, binding and injecting the AMR gene it is carrying (5). If this gene is successfully integrated into the bacterial chromosome (6), this creates a new transductant bacterium carrying this AMR gene (7). Note that the transduced bacterial DNA could also be a plasmid, in which case it would circularize instead of integrating into the chromosome of the transductant bacterium. The figure is not to scale.

There are two main types of transduction; here, we focus on generalized transduction, which occurs during the phage lytic cycle when nonphage genome DNA is mistakenly packaged in a new phage particle (Fig. 1). The resulting transducing phage released upon lysis can then inject this genetic material into another bacterium. The second type of transduction, specialized transduction, relies on lysogeny, during which sections of bacterial DNA adjacent to the prophage integration site may be transferred upon excision of the prophage (8, 9). Generalized transduction is currently often dismissed as too rare to be significant, yet it is likely a substantial contributor to AMR spread, as it is a common mechanism for the transfer of plasmids, major vectors of AMR genes (2). There are currently no estimates or work quantifying rates of transduction of AMR genes under various conditions. Previous reviews have highlighted the necessity to further investigate the potential impact of transduction in the context of phage therapy, where phage are used as antimicrobial agents against bacteria (10–13).

Mathematical models have been used to gain insights into phage predation dynamics that cannot be obtained solely with experimental work, such as rates of predation and optimal conditions for phage to clear bacteria (14). Such models typically assume a density-dependent interaction, with new phage infections calculated as the number of susceptible bacteria multiplied by the number of phage and an adsorption constant (14–16). This approach has limitations, as density-dependent models have failed to predict equilibria observed under some *in vitro* conditions between phage and Escherichia coli (17). Moreover, phage and bacterial replication are likely linked, as they both rely on the bacterial machinery; phage predation may slow as bacteria reach stationary phase (14, 17–23). However, this is a feature that is not commonly included in mathematical models of phage-bacteria dynamics (14). Finally, models often only rely on data of phage-bacteria interactions measured once per day or for a few hours (17–19, 24). A current lack of detailed data means that capturing these underlying dynamics, which occur in less than an hour, has not yet been possible.

To the best of our knowledge, only three modeling studies have included transduction of AMR genes (25–27). All three modeled complex environments, including resistance to phage, antibiotics, and both lytic and lysogenic cycles. This complexity, combined with the fact that these studies were not paired with complementary *in vitro* or *in vivo* data, means that they relied on assumptions and previously published estimates instead of parameter values derived from a single environment and set of conditions. This limits the wider reliability of conclusions made using these models (12).

In this article, we investigate the dual nature of phage dynamics using the clinically relevant bacteria methicillin-resistant Staphylococcus aureus (MRSA) (28). Transduction is the main mechanism of horizontal gene transfer driving evolution for these bacteria (29), and phage therapy is currently being investigated to treat MRSA infections (30, 31). We aim to clarify the joint dynamics of predation and generalized transduction between MRSA and phage by generating novel *in vitro* data, identifying biologically plausible hypotheses that may explain the dynamics seen, and developing mathematical models to test the validity of these hypotheses in our system.

## RESULTS

### Transduction and phage predation dynamics *in vitro*.

We focused on two laboratory strains of Staphylococcus aureus, each resistant to either erythromycin (referred to as B_E_) or tetracycline (B_T_). Under our experimental conditions, the antimicrobial resistance (AMR) genes can only be transferred between bacteria by generalized transduction mediated by exogenous phage. Transduction of either AMR gene to the other strain will result in the formation of double-resistant progeny (DRP; also referred to as B_ET_).

**FIG 2:**
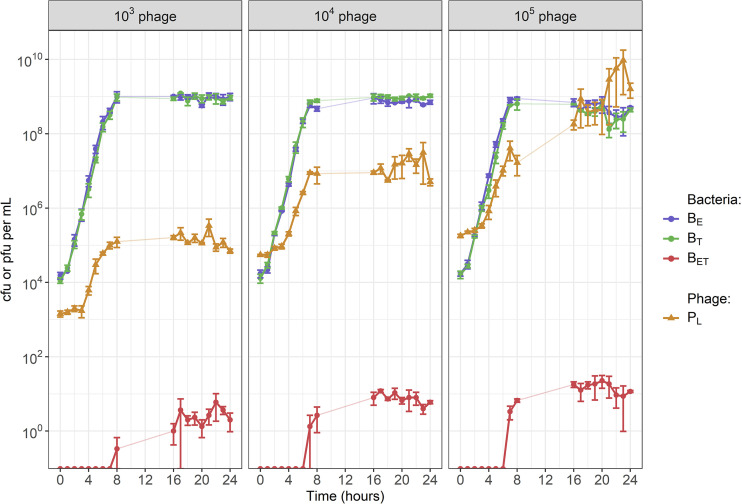
Starting concentration of exogenous phage 80α affected the equilibrium values of phage and bacteria in our cocultures. The starting concentration of both single-resistant S. aureus parent strains (B_E_ for erythromycin and B_T_ for tetracycline) was 10^4^ CFU per mL. Each panel shows the results with a different starting concentration of exogenous phage (P_L_): either 10^3^, 10^4^, or 10^5^ plaque-forming units (PFU) per mL. We detected double-resistant progeny (B_ET_) as early as 7 h, indicating that transduction occurred rapidly. Error bars indicate means ± standard errors from 3 experimental replicates. There are no data for the time period of 9 h to 15 h.

We conducted a coculture with only the two single-resistant strains and exogenous phage (P_L_) capable of generalized transduction. We detected DRP (B_ET_) as early as 7 h in our cocultures, indicating that transfer of AMR genes by generalized transduction had occurred (Fig. 2). B_ET_ numbers remained below 100 CFU/ml after 24 h but were consistently generated in each of our experimental replicates. Colonies of DRP were screened using PCR to confirm that they contained both resistance genes *erm*(B) and *tet*(K) and had not instead gained resistance to either antibiotic by mutation (see Fig. S1 in the supplemental material).

10.1128/msystems.00135-22.1 FIG S1 Confirmation of DRP by polymerase-chain reaction. Five single colonies were sampled from a double antibiotic plate (1 to 5) containing bacteria plated after 24 h of coculture started only with single-resistant parent strains (E and T) and exogenous phage. L, ladder; E, erythromycin resistance gene [*erm*(B)]; T, tetracycline resistance gene [*tet*(K)]. Download FIG S1, TIF file, 0.6 MB. Copyright © 2022 Leclerc et al. 2022 Leclerc et al. https://creativecommons.org/licenses/by/4.0/ This content is distributed under the terms of the Creative Commons Attribution 4.0 International license.

The starting concentration of exogenous phage affected whether phage and bacteria were able to reach an equilibrium and coexist without increasing or decreasing in our cocultures (Fig. 2). With a starting concentration of either 10^3^ or 10^4^ PFU/mL (equivalent to multiplicities of infection [MOI] of 0.1 or 1, defined as a starting ratio of phage to bacteria [32]), lytic phage reached a steady state after 8 h (at approximately 10^5^ PFU/mL for a starting concentration of 10^3^ and 10^7^ PFU/mL for 10^4^). In both cases, bacteria replicated for 8 h before reaching a steady state around 10^9^ CFU/mL, similar to what was seen in the absence of exogenous phage (Fig. S2). With a starting phage concentration of 10^5^ PFU/mL (MOI of 10), we did not see an equilibrium between phage and bacteria, as phage numbers kept increasing to 10^10^ PFU/mL by 24 h, and bacterial numbers started decreasing after 20 h. The data sets are shown overlaid in Fig. S3.

10.1128/msystems.00135-22.2 FIG S2 Growth curves for bacteria in the absence of exogenous phage. *B_E_*, bacteria resistant to erythromycin; *B_T_*, bacteria resistant to tetracycline; *B*_ET_, bacteria resistant to both erythromycin and tetracycline. Solid lines correspond to *in vitro* data, and dashed lines to the model output generated using the median values of the parameter distributions obtained by model fitting. Shaded areas indicate error obtained by resampling the model results from a Poisson distribution 10 times. Download FIG S2, TIF file, 0.2 MB. Copyright © 2022 Leclerc et al. 2022 Leclerc et al. https://creativecommons.org/licenses/by/4.0/ This content is distributed under the terms of the Creative Commons Attribution 4.0 International license.

10.1128/msystems.00135-22.3 FIG S3 Transduction coculture datasets overlaid. The starting concentration of both single-resistant S. aureus parent strains (*B_E_* for erythromycin and B_T_ for tetracycline) is 10^4^ CFU per mL. The starting concentration of exogenous phage 80α (P_L_) is either 10^3^ (solid lines), 10^4^ (dashed), or 10^5^ (dotted) PFU per mL. Error bars indicate means ± standard errors from 3 experimental replicates. There is no data for the time period 9 to 15 h. Download FIG S3, TIF file, 0.6 MB. Copyright © 2022 Leclerc et al. 2022 Leclerc et al. https://creativecommons.org/licenses/by/4.0/ This content is distributed under the terms of the Creative Commons Attribution 4.0 International license.

### Absence of lysogeny in our coculture.

The phage we used in our experiments is 80α, a well-known generalized transducing phage. It has also been reported as a temperate phage, which means that it may undergo lysogeny and integrate in the bacterial chromosome as a prophage (33). This would grant lysogenic immunity to the bacteria, preventing further lytic infection by 80α and potentially explaining why bacterial and phage densities reached steady states in our experiments (Fig. 2).

**FIG 3:**
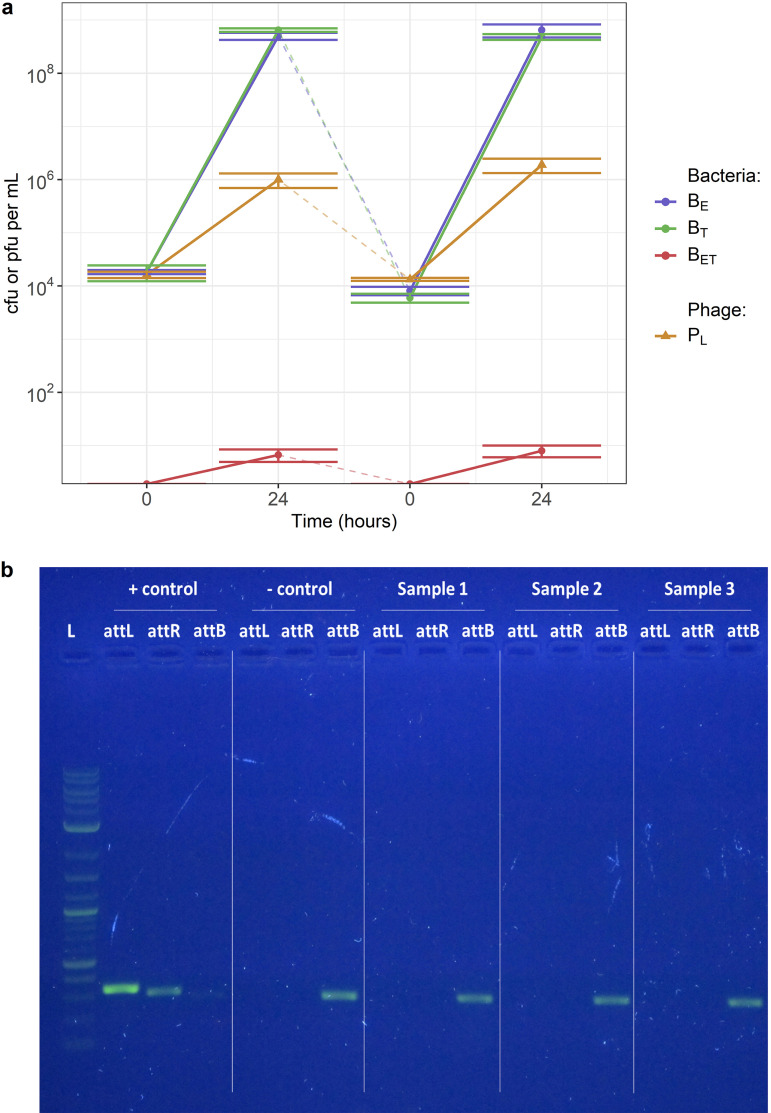
80α lysogeny does not occur at a detectable level in our coculture. (a) Cocultures with bacteria not exposed or previously exposed to phage. The starting concentration of both single-resistant S. aureus parent strains (B_E_ for erythromycin and B_T_ for tetracycline) was 10^4^ CFU per mL, and the starting concentration of exogenous phage 80α (P_L_) was 10^4^ PFU per mL. double-resistant progeny (B_ET_) are generated by transduction. The initial coculture was diluted in fresh media after 24 h to form a new coculture with bacteria previously exposed to phage. Phage were added in the new coculture to reach a concentration of 10^4^ PFU/mL. Error bars indicate means ± standard errors, from 3 experimental replicates. (b) Confirmation of absence of detectable lysogeny by polymerase chain reaction. DNA was extracted from the cocultures after 24 h. S. aureus RN4220 strains lysogenic and nonlysogenic for 80α were used as positive and negative controls. L, ladder; *attL*, left prophage junction; *attR*, right prophage junction; *attB*, bacterial insertion site. Detection of *attL* and *attR* indicates that prophage are present in the DNA, while detection of *attB* indicates the presence of bacteria not lysogenic for 80α.

To investigate whether this was a potential mechanism, we initiated cocultures either with stock bacteria or bacteria exposed to phage during a previous coculture. We did not see any difference in phage and bacterial numbers after 24 h regardless of whether or not the bacteria had been previously exposed to phage, suggesting that lysogenic immunity has not been substantially gained by bacteria over 24 h (Fig. 3a).

In addition, we extracted DNA from 1 mL of cocultures after 24 h and conducted PCRs targeting the prophage junctions (*attL* and *attR*) and bacterial insertion site (*attB*) with a positive control of a strain lysogenic for 80α. Our DNA extraction and PCR protocol mean that the detection limit for our protocol is a frequency of at least 3.3 × 10^−8^ lysogenic per nonlysogenic bacteria after 24 h of coculture (see Materials and Methods for details). Only the intact bacterial insertion site was detected in our samples, indicating an absence of prophage in our bacteria above this detection limit (Fig. 3b).

Another concern linked to lysogeny we must address is that, if lysogeny did occur, the movement of the resistance genes *tet*(K) and *erm*(B) could have occurred by specialized instead of generalized transduction. However, this is unlikely to be the case in our system, since specialized transduction can only lead to transfer of genes adjacent to the integrated prophage (8, 9). This adjacency limitation also applies to lateral transduction, a type of specialized transduction reported for 80α leading to higher rates of transfer for DNA located downstream of the insertion site (34). This condition of proximity to the insertion site is not met in our system. The tetracycline resistance marker *tet*(K) is located on a plasmid where 80α cannot integrate, preventing specialized and lateral transduction. As for the erythromycin resistance marker *erm*(B), the distance between the location of this gene on the chromosome (bp position 2126759 [35]) and the 80α integration site (next to the *rpmF* gene [33], bp position 1122198 [35]) suggests specialized and lateral transduction are unlikely.

Overall, these results suggest that after 24 h the frequency of lysogenic per nonlysogenic bacteria is less than 3.3 × 10^−8^ in our coculture; hence, it is reasonable to exclude any dynamics relating to lysogeny and specialized or lateral transduction in our analysis and model below. Therefore, phage lysis and generalized transduction are likely the main mechanisms shaping phage-bacteria interactions in our coculture.

### Bacterial growth estimates in the absence of exogenous phage.

When grown together in the absence of exogenous phage, single- and double-resistant bacteria replicated exponentially and reached stationary phase after 8 h at 10^9^ CFU per mL (Fig. S2).

B_E_ did not show a significant fitness cost relative to B_T_ over 24 h of growth (mean relative fitness, 1.02; standard deviations [SD], 0.03). The DRP B_ET_ did not show a significant fitness cost relative to either single-resistant parent strain (mean relative fitness to B_E_, 0.96; SD 0.06; mean relative fitness to B_T_, 0.98; SD 0.03).

We obtained maximum growth rate estimates by fitting a logistic growth model to the *in vitro* data.

The median estimated maximum growth rates were 1.61 for B_E_ (95% credible interval, 1.59 to 1.63), 1.51 for B_T_ (1.49 to 1.53), and 1.44 for B_ET_ (1.42 to 1.47), with a total carrying capacity of 2.76 × 10^9^ CFU/mL (2.61 × 10^9^ to 2.98 × 10^9^).

### Investigation of possible phage-bacteria interactions using a flexible modeling framework. (i) Model structure.

**FIG 4:**
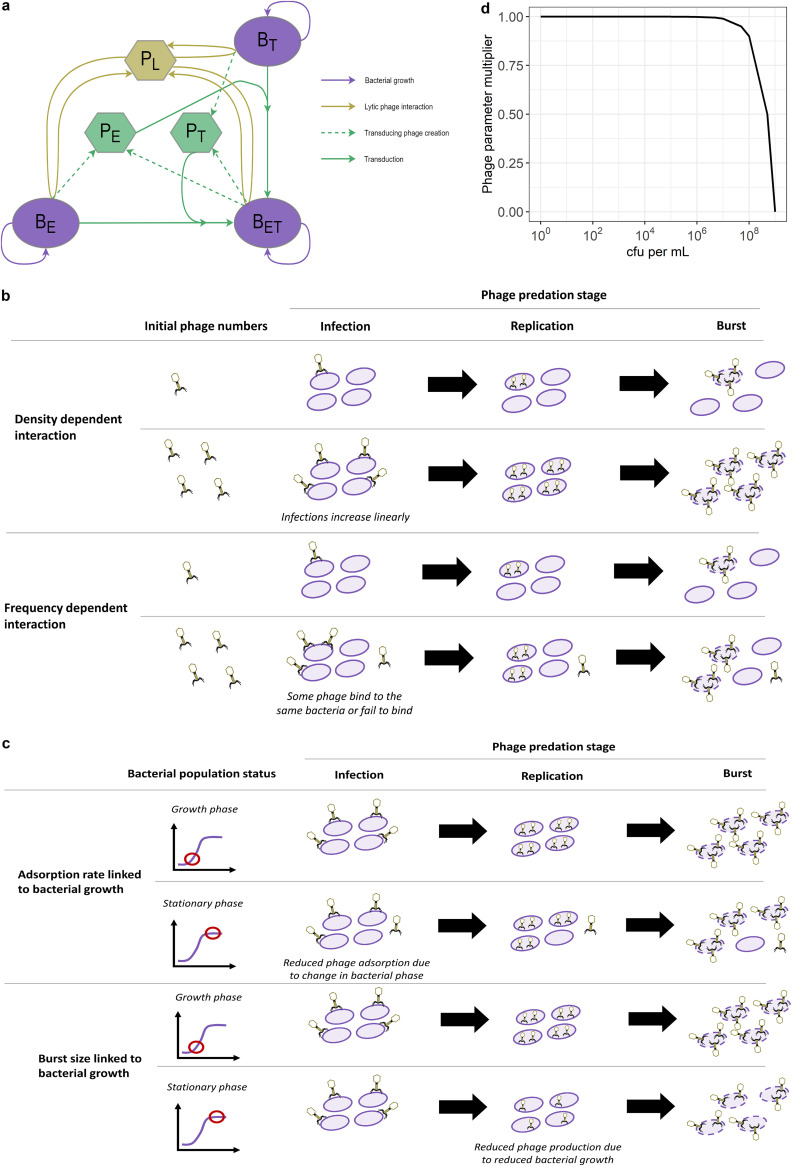
Phage predation and generalized transduction model diagram and different phage-bacteria interactions considered. (a) Model diagram. Each bacteria strain (B_E_, resistant to erythromycin; B_T_, resistant to tetracycline; B_ET_, resistant to both) can replicate (purple). The lytic phage (P_L_) multiply by infecting a bacterium and bursting it to release new phage (gold). This process can create transducing phage (P_E_ or P_T_) carrying a resistance gene [*erm*(B) or *tet*(K), respectively] taken from the infected bacterium (green). These transducing phage can then generate new DRP (*B*_ET_) by infecting the bacterial strain carrying the other resistance gene (green). (b) Phage predation in the model is either density- or frequency-dependent. (Top) With a density-dependent interaction, the number of infections scales linearly with the number of phage and bacteria. (Bottom) A frequency-dependent interaction illustrates that some phage may not infect a bacterium or that multiple phage may infect the same bacterium. (c) Phage predation in the model can decrease as bacterial growth decreases. (Top) A change in bacterial growth phase can affect surface receptors, leading to a reduced phage adsorption rate. (Bottom) Since phage replication relies on bacterial processes, reduced bacterial growth can translate into reduced phage burst size. (d) Proposed function linking phage predation parameters to bacterial growth. This shows the multiplier applied to decrease phage parameters as the bacterial population increases toward carrying capacity, equivalent to a decrease in bacterial growth. Here, the carrying capacity is 2.76 × 10^9^ CFU/mL, estimated from our data.

We designed a mathematical model to reproduce the *in vitro* phage-bacteria dynamics, including generalized transduction of resistance genes. During our experiment, our coculture contained up to three strains of bacteria: the two single-resistant parents (B_E_ and B_T_) and the DRP (B_ET_). Although we were only able to count lytic phage (P_L_), based on the biology of generalized transduction (Fig. 1) we know that there were also transducing phage carrying either the erythromycin resistance gene (P_E_) or the tetracycline resistance gene (P_T_). Since we did not detect any evidence of 80α lysogeny in our coculture after 24 h, we did not include this feature in the model. The corresponding model diagram is shown in Fig. 4a. The complete model equations can be found in Materials and Methods.

Using this modeling framework, we explored a combination of different phage-bacteria interactions, described below (Fig. 4b and c). By fitting the models to our experimental data, we could rule out certain interactions and suggest the best model to reproduce the phage-bacteria dynamics seen *in vitro*.

### (ii) First phage-bacteria interaction: density versus frequency-dependent phage predation.

The most common approach to model phage-bacteria dynamics is to assume that phage predation is density-dependent (14). This means that, over one time step, the number of phage infecting bacteria and the number of bacteria infected by phage are both equal to the product of the number of bacteria (*B*), phage (*P*), and phage adsorption rate (β), as shown in equation 1.

(1) B×P×β

The density-dependent interaction implies that the number of new infections scales linearly with the number of phage and bacteria (Fig. 4b). Therefore, even if we keep a constant number of phage, increasing bacteria numbers always leads to a linear increase in the estimated number of new infections. Although this simplification is useful and holds for a range of values, it has been suggested that it is not biologically realistic for small numbers of phage or bacteria, since in reality one phage can only infect one bacterium over one time step (17).

To overcome these issues, we consider an alternative interaction, where phage predation is frequency-dependent (36). This accounts for the fact that one phage does not necessarily always lead to one infection. For example, phage may sometimes fail to bind to bacteria, or multiple phage may bind to the same bacterium (32) (Fig. 4b). Importantly, this mathematical interaction guarantees that, at any given time point, the number of phage infecting bacteria and the number of bacteria infected by phage can never be greater than the total number of phage or bacteria in the system. Over one time step, the proportion of phage infecting any bacteria (λ) is defined by equation 2.

(2) λ=[1−exp (−β×B)]

Similarly, the proportion of bacteria being infected by at least one phage (φ) is calculated with equation 3.

(3) $$\text{φ}=\left[1-\text{exp }\left(-\frac{\lambda \times P}{B}\right)\right]$$

### (iii) Equilibrium analyses for the density- and frequency-dependent models.

Despite these being common methods to represent phage-bacteria interactions in mathematical models, previous analyses have suggested that the density- and frequency-dependent interactions alone cannot capture the equilibrium levels we and others have seen (18, 37). We explore this in the context of our own *in vitro* data using equilibrium analyses.

Assuming that transduction and the phage latent period are negligible, a simplified model representing phage predation as a density-dependent process is shown in equations 4 and 5.

(4) $$\frac{\mathrm{dB}}{\mathrm{dt}}={\text{μ}}_{\text{max}}\times B\times (1-\frac{B}{B_{\text{max}}})-B\times P\times \beta$$

(5) $$\frac{\mathrm{dP}}{\mathrm{dt}}=B\times P\times \beta \times \delta \text{′}-\gamma \times P$$

Where μ_max_ is the maximum bacterial growth rate, *B*_max_ is the carrying capacity, β is the phage adsorption rate, γ is the phage decay rate, and δ is the phage burst size, with δ′ equal to δ − 1. To solve for equilibrium (i.e., $\frac{\mathrm{dB}}{\mathrm{dt}}=\frac{\mathrm{dP}}{\mathrm{dt}}=0$), equations 4 and 5 can be rewritten as equations 6 and 7.

(6) $${\text{μ}}_{\text{max}}\times B\times (1-\frac{B}{B_{\text{max}}})-B\times P\times \beta =0$$

(7) B×P×β×δ′−γ×P=0

Since we are interested in an equilibrium with the condition that there are still bacteria and phage in the environment (i.e., *B* ≠ 0 and *P* ≠ 0), we can divide equations 6 and 7 by *B* and *P*, respectively, to obtain equations 8 and 9. These must hold true for there to be a nonzero bacteria and phage population at equilibrium.

(8) $${\text{μ}}_{\text{max}}\times (1-\frac{B}{B_{\text{max}}})-\;P\times \beta =0$$

(9) B×β×δ′−γ=0

We then obtain equations 10 and 11 by rearranging equations 8 and 9 to give expressions for *P* and *B* at equilibrium.

(10) $$P=\;\frac{{\text{μ}}_{\text{max}}}{\beta }\times (1-\frac{B}{B_{\text{max}}})$$

(11) $$B=\frac{\gamma }{\beta \times \delta \text{′}}$$

In our experiment with a starting phage concentration of 10^4^ PFU/mL, after 24 h the bacterial concentration was approximately 10^9^ CFU/mL and the phage concentration was 10^5^ PFU/mL. If we replace the corresponding terms in equations 10 and 11 with these values, alongside the carrying capacity (2.8 × 10^9^) and average of the growth rates estimated (1.52), we obtain equations 12 and 13.

(12) $$10^{5}=\frac{1.52}{\beta }\times (1-\frac{10^{9}}{2.8\times 10^{9}})$$

(13) $$10^{9}=\frac{\gamma }{\beta \times \delta \text{′}}$$

Rearrangement of equation 12 leads to a solution for phage adsorption (β) (equation 14).

(14) $$\beta \;=9.77\times 10^{-6}\;\approx \;10^{-5}$$

Substituting this into equation 13 leads to a value for phage decay rate (γ) (equation 15).

(15) $$\gamma =\;10^{9}\times 10^{-5}\times \delta \text{′ }\approx \;10^{4}\times \delta \text{′}$$

This gives rise to the condition that the phage decay rate, γ, must be approximately 10^4^ times greater than the burst size, δ′. As at least one phage must be released upon bursting, the burst size, δ′, is greater than 1. However, the phage decay rate, γ, which represents the proportion of phage inactivated during one time step, must be less than 1; hence, this is impossible.

As for the frequency-dependent interaction, a simplified model of this process is shown in equations 16 and 17.

(16) $$\frac{\mathrm{dB}}{\mathrm{dt}}={\text{μ}}_{\text{max}}\times (1-\frac{B}{B_{\text{max}}})\times (B-\text{φ}\times B)-\text{φ}\times B$$

(17) $$\frac{\mathrm{dP}}{\mathrm{dt}}=\text{φ}\times B\times \delta -\lambda \times P-\text{ γ}\times P$$

With the condition that $\frac{\mathrm{dB}}{\mathrm{dt}}=0$, equation 16 can be rewritten as equation 18. This condition must hold for there to be a nonzero equilibrium.

(18) $$\text{φ}={\text{μ}}_{\text{max}}\times (1-\frac{B}{B_{\text{max}}})\times (1-\text{φ})$$

Using our equilibrium values (*B* = 10^9^, *P* = 10^5^, *B*_max_ = 2.8 × 10^9^, μ_max_ = 1.52), this is equivalent to equation 19.

(19) $$\text{φ}=1.52\times \left(1-\text{φ}\right)\times (1\;-\frac{10^{9}}{2.8\times 10^{9}})$$

Equation 19 is solved to obtain a value for φ (equations 20 to 23).

(20) φ=0.97×(1−φ)

(21) φ=0.97−0.97×φ

(22) 1.97×φ=0.97

(23) φ=0.49

Using our equilibrium values and this value of 0.49 for φ, equation 3 can be rewritten as equation 24.

(24) $$0.49=1-\text{exp }\left(-\frac{\text{λ}\times 10^{5}}{10^{9}}\right)$$

Equation 24 is solved to obtain a value for λ (equations 25 to 29).

(25) $$0.49=1-\text{exp }\left(-\frac{\text{λ}}{10^{4}}\right)$$

(26) $$0.51=\text{exp }\left(-\frac{\text{λ}}{10^{4}}\right)$$

(27) $$\text{ln}(0.51)=\;-\frac{\text{λ}}{10^{4}}$$

(28) $$-\text{ln}(0.51)\times 10^{4}=\lambda$$

(29) λ ≈ 6,733

Therefore, λ must be approximately equal to 6,733 to obtain a nonzero equilibrium, as seen in our *in vitro* data with our model-fitted values (biologically plausible ones). However, the definition of λ according to equation 2 implies that λ is <1. Therefore, this is impossible.

Even though these analyses rely on a simplified set of equations, using realistic parameter values we have shown that a nonzero equilibrium, as we have seen *in vitro*, cannot be reproduced using models with only a density- or frequency-dependent interaction. Instead, phage-bacteria coexistence may be explained by variations in phage predation parameters depending on bacterial resource availability or bacterial growth rate (14, 17–22). However, to the best of our knowledge a simple mathematical expression linking phage predation to bacterial growth has not yet been developed.

### (iv) Second phage-bacteria interaction: dependence of phage predation on bacterial growth.

Here, we consider that a decrease in bacterial growth as bacteria reach stationary phase could first affect the phage adsorption rate, β, due to changes in receptors on bacterial surfaces, which affect opportunities for phage to bind (Fig. 4c). Second, this could affect phage production and, thus, burst size, δ, as phage replication relies on bacterial processes and may decrease when bacterial growth slows down (Fig. 4c). Using a single phage predation multiplier, with the same principle of logistic growth applied to bacteria, we allow either or both β and δ to decrease as bacterial growth decreases in our model (equations 30 and 31).

(30) $$\beta ={\text{β}}_{\text{max}}\times \left(1-\frac{B}{B_{\text{max}}}\right)$$

(31) $$\delta ={\text{δ}}_{\text{max}}\times \left(1-\frac{B}{B_{\text{max}}}\right)$$

These equations imply that as bacterial population size increases toward carrying capacity (*B*_max_), phage parameters will be reduced (Fig. 4d).

### Identification of the best-fitting phage-bacteria interactions to reproduce the *in vitro* dynamics.

Overall, we considered 6 different models, either density or frequency dependent and with either or both the phage adsorption rate and burst size linked to bacterial growth. Note that we did not include a phage decay rate in these models, as this did not affect the dynamics of the system over 24 h, for a wide range of decay rates (Fig. S4).

10.1128/msystems.00135-22.4 FIG S4 Model results are not affected by phage decay rate over a wide range of values. Previous estimates of phage decay rate per hour are between 10^−3^ *in vitro* and up to 10^−1^ *in vivo* (38). Models are either density- or frequency-dependent, with either or both the phage adsorption rate and burst size linked to bacterial growth. Download FIG S4, TIF file, 1.0 MB. Copyright © 2022 Leclerc et al. 2022 Leclerc et al. https://creativecommons.org/licenses/by/4.0/ This content is distributed under the terms of the Creative Commons Attribution 4.0 International license.

**FIG 5:**
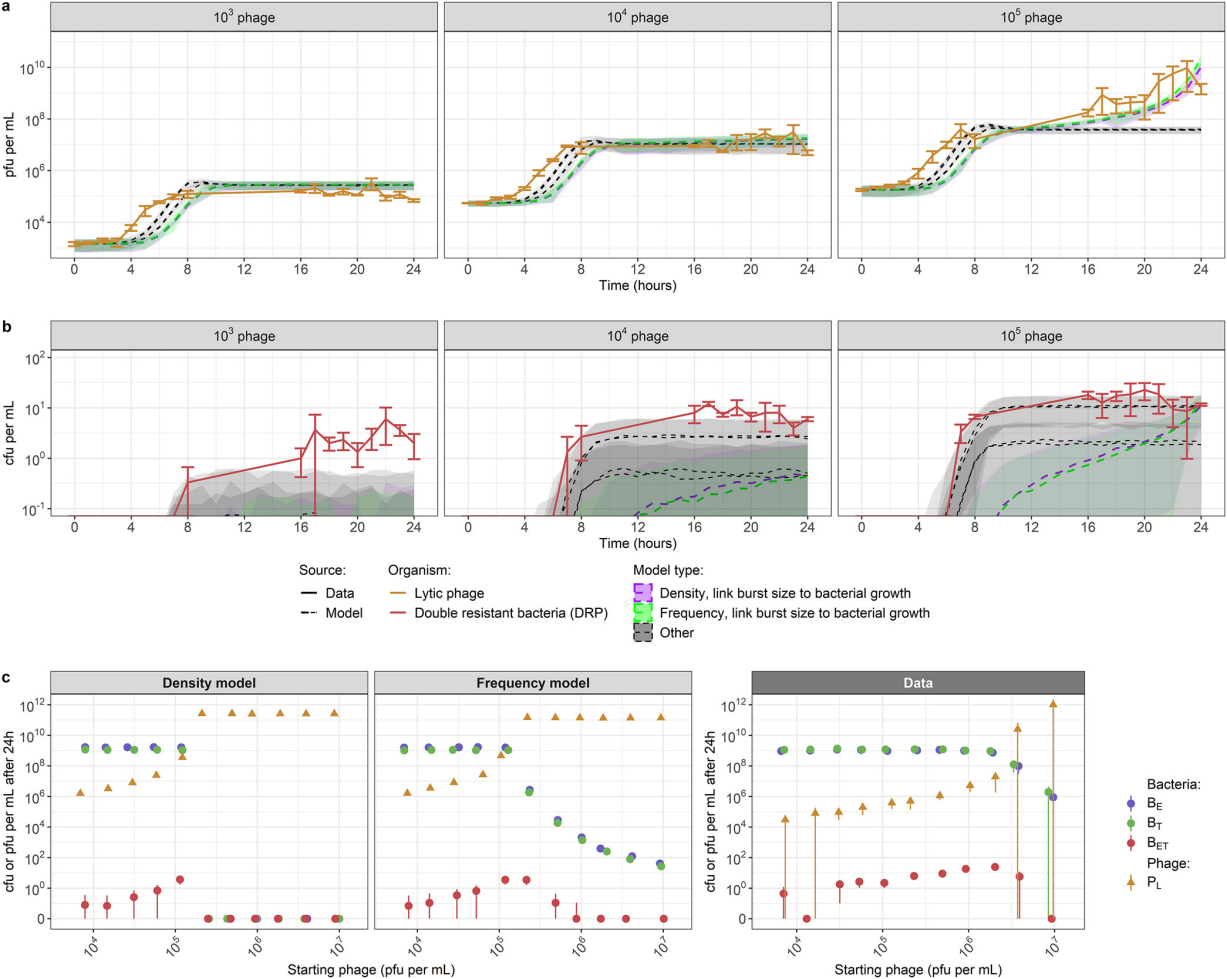
Accuracy of the best-fitted models to reproduce *in vitro* phage-bacteria dynamics. (a and b) The models with only phage burst size linked to bacterial growth are the most accurate to reproduce *in vitro* trends in lytic phage (a) and double-resistant bacteria (b) numbers, starting from a bacterial concentration of 10^4^ CFU/mL and varying phage concentrations. All models (dashed lines) can reproduce the trends seen *in vitro* when phage are started at 10^3^ or 10^4^ PFU/mL (data in solid lines), but only the models with just the phage burst size linked to bacterial growth (colored model output) can reproduce the trend seen when phage are started at 10^5^ PFU/mL. Other models (gray) have only the phage adsorption rate linked to bacterial growth or both the phage adsorption rate and burst size. Models are fitted to the 10^3^ and 10^5^ data and tested with the 10^4^ data. Parameter values used are the median fitted values (Table 1). Shaded areas indicate standard deviations generated from Poisson resampling of model results. Error bars for the data (solid lines) indicate means ± standard errors from 3 experimental replicates. (c) When further testing fitted model dynamics starting from 10^6^ CFU/mL bacteria and varying phage concentrations, the density-dependent model incorrectly predicts bacterial extinction, while the frequency-dependent model reproduces the trend but not the exact values of the 24 h data. In the coculture used to generate the data, each single-resistant parent strain (B_E_ and B_T_) is added at a starting concentration of 10^6^ CFU/mL, and no DRP (B_ET_) are initially present. The starting concentration of lytic phage (P_L_) varies (*x* axis). Points indicate mean results and are each slightly shifted horizontally to facilitate viewing. Error bars indicate either means ± standard deviation for the models (left/center) or means ± standard errors for the data (right). Parameter values used are the median fitted values (Table 1).

All models successfully reproduced the trends seen *in vitro* when the phage were started at either 10^3^ or 10^4^ PFU/mL (Fig. 5a and b). However, only the two models where only phage burst size decreases as the bacterial population approaches carrying capacity could reproduce the increase in phage numbers seen in the later hours of the 10^5^ PFU/mL data set, despite all models having been fitted to this data set (Fig. 5a and b). This was confirmed by calculating the average deviance information criterion (DIC) value for the models, which favors best-fitting models while penalizing more complex models (i.e., those with more parameters) (38). The two models where only phage burst size decreases as the bacteria population approaches carrying capacity had the lowest DIC values, indicating that they were the better-fitting models (Table 1).

**TABLE 1:** Estimated parameter values from fitting to *in vitro* data^*a*^

Interaction type	Adsorption rate linked to growth	Burst size linked to growth	Adsorption rate β (phage^−1^ bacteria^−1^ h^−1^)	Burst size δ (phage)	Transducing phage proportion α (proportion of burst size)	Phage latent period τ (h)	DIC
Density dependent	Yes	No	4.5 × 10^−9^ (4.1 × 10^−9^; 5.0 × 10^−9^)	12 (10; 14)	3.1 × 10^−8^ (1.5 × 10^−8^; 5.8 × 10^−8^)	0.64 (0.55; 0.73)	610
	No	Yes	1.6 × 10^−10^ (1.5 × 10^−10^; 1.7 × 10^−10^)	79 (72; 86)	1.4 × 10^−8^ (1.1 × 10^−8^; 1.7 × 10^−8^)	0.65 (0.62; 0.69)	63
	Yes	Yes	4.3 × 10^−9^ (3.9 × 10^−9^; 4.6 × 10^−9^)	43 (37; 49)	1.2 × 10^−8^ (6.4 × 10^−9^; 2.3 × 10^−8^)	0.93 (0.86; 0.99)	298
Frequency dependent	Yes	No	5.1 × 10^−9^ (3.7 × 10^−9^; 6.7 × 10^−9^)	10 (8; 12)	3.1 × 10^−7^ (2.3 × 10^−7^; 4.3 × 10^−7^)	0.60 (0.50; 0.69)	680
	No	Yes	2.3 × 10^−10^ (2.1 × 10^−10^; 2.4 × 10^−10^)	76 (70; 83)	1.0 × 10^−8^ (8.5 × 10^−9^; 1.4 × 10^−8^)	0.72 (0.69; 0.77)	0
	Yes	Yes	4.7 × 10^−9^ (3.8 × 10^−9^; 5.8 × 10^−9^)	31 (26; 37)	1.7 × 10^−7^ (1.3 × 10^−7^; 2.1 × 10^−7^)	0.88 (0.79; 0.96)	370

a Values show median and 95% credible intervals for posterior distributions. Parameter units are indicated in parentheses. Fitting was performed using the Markov chain Monte Carlo Metropolis–Hastings algorithm and the data from the coculture with a starting bacterial concentration of 10^4^ CFU/mL and phage concentration of 10^3^ and 10^5^ PFU/mL. DIC, deviance information criteria. A smaller DIC indicates better model fit. DIC values are relative to the smallest DIC calculated, which is for the frequency-dependent model with only burst size linked to bacterial growth (line 5).

Our initial experiments considered the dynamics over 24 h for various phage starting concentrations. To test the ability of our model to recreate the dynamics under changing bacterial levels, we replicated our transduction coculture experiments with starting concentrations of 10^6^ CFU/mL bacteria instead of 10^4^ CFU/mL, varying the starting phage concentration between 10^4^ and 10^6^ PFU/mL and measuring bacterial and phage numbers after 24 h of coculture. We then used the estimated parameter values (Table 1) to try to reproduce these 24 h numbers of bacteria and phage.

Increasing the starting phage concentration led to an increase in the number of phage after 24 h (Fig. 5c). For a starting phage concentration between 10^4^ and 10^6^ PFU/mL, increasing starting phage numbers did not affect single-resistant parents B_E_ and B_T_ numbers after 24 h but led to a progressive increase in DRP B_ET_ numbers. Increasing starting phage numbers above 10^6^ PFU/mL caused bacteria numbers after 24 h to decrease.

Using the estimated parameter values (Table 1) with the model where only burst size is linked to bacterial growth, we see that the density-dependent model cannot reproduce these dynamics, as it predicts that bacteria become extinct rapidly (Fig. 5c). The frequency-dependent model can reproduce these trends but fails to recreate the exact same numbers of phage and bacteria, predicting a decline in bacterial levels when the starting phage concentration increases above 10^5^ PFU/mL, a lower threshold than that seen in the data (Fig. 5c). The same overall trends are seen for the models where only the adsorption rate is linked to bacterial growth, or both adsorption rate and burst size (Fig. S5).

10.1128/msystems.00135-22.5 FIG S5 Model performance in reproducing the 24 h data values for different starting concentrations of phage with different links between phage predation and bacterial growth rate. (A) Phage adsorption rate decreases as bacterial growth rate decreases. (B) Phage burst size and adsorption rate decrease as bacterial growth rate decreases. Phage predation is either density- or frequency-dependent in the models. Model parameters are those estimated for the corresponding model as shown in Table 1. In the coculture used to generate the data, each single-resistant parent strain (*B_E_* and *B_T_*) is added at a starting concentration of 10^6^ CFU/mL, and no DRP (*B*_ET_) are initially present. The starting concentration of lytic phage (P_L_) varies (*x* axis). Download FIG S5, TIF file, 1.2 MB. Copyright © 2022 Leclerc et al. 2022 Leclerc et al. https://creativecommons.org/licenses/by/4.0/ This content is distributed under the terms of the Creative Commons Attribution 4.0 International license.

### Analysis of phage predation and transduction dynamics.

**FIG 6:**
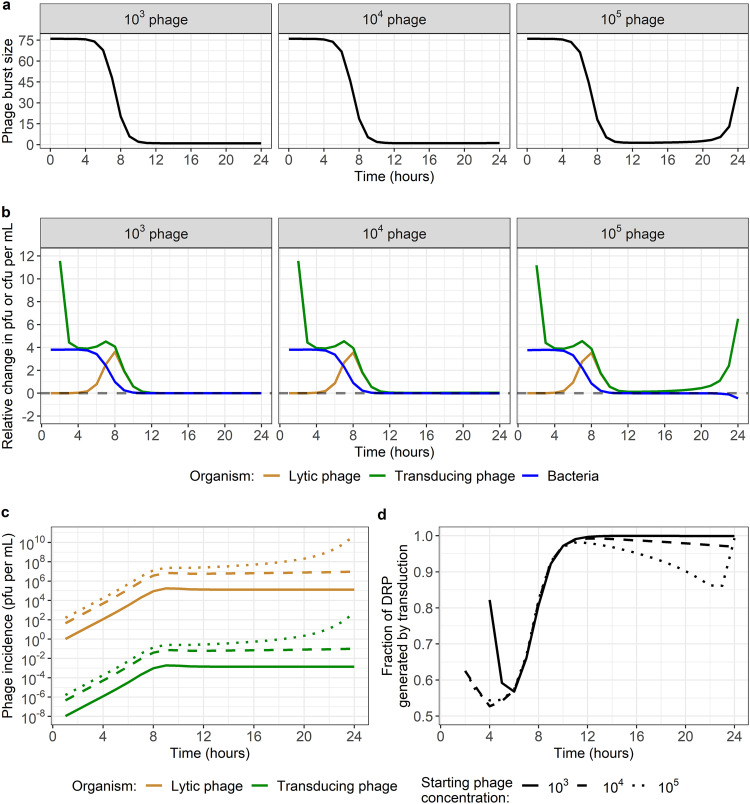
Underlying phage and bacteria dynamics generated by the best-fitting frequency-dependent model with burst size linked to bacterial growth. Model parameters are the median estimates from model fitting (Table 1). (a) Phage burst size over time by starting phage concentration. As bacteria reach stationary phase after 8 h, phage burst size decreases. In the 10^5^ data set, we see that burst size is predicted to increase again after 20 h. This is due to bacterial numbers decreasing as bacteria are being lysed by phage. (b) Relative change in phage and bacterial numbers over time by starting phage concentration. The number of new phage generated at each time step increases (positive value) until bacteria reach stationary phase around 8 h. This applies to lytic and transducing phage. In the 10^5^ data set, phage keep increasing after 10 h, eventually causing a decrease in bacterial numbers (negative value), which translates into a further acceleration in the increase in phage numbers due to the increased burst size (Fig. 5a). After 8 h, the relative changes in lytic and transducing phage numbers are identical. (c) Incidence of lytic (gold) and transducing (green) phage over time by starting phage concentration (line type). For any data set and time point, there is approximately 1 new transducing phage generated for each 10^8^ new lytic phage. (d) Fraction of DRP generated by transduction each hour over time by starting phage concentration (line type). DRP generation always occurs predominantly by transduction rather than by growth of already existing DRP. Note that the time at which DRP are first generated varies by starting phage concentration.

Parameter estimates for our best-fitting model (with a frequency-dependent interaction and a link between phage burst size and bacterial growth only) suggest that the adsorption rate is 2.3 × 10^−10^ (95% credible interval, 2.1 × 10^−10^ to 2.4 × 10^−10^), which is the smallest estimate from the models (Table 1). On the other hand, the estimated burst size is relatively large, at 76 (70 to 83) phage, and is higher than a previous *in vitro* estimate for 80α of 40 (39). However, due to the decrease in burst size when bacteria are in stationary phase, we expect that this number would change depending on the conditions under which it is measured (Fig. 6a). Finally, the estimated latent period of 0.72 h (0.69 to 0.77) is slightly longer than a previous *in vitro* estimate of 0.67 h (39). Regarding the other models, we note some biologically unlikely parameter estimates that further suggest that these models are inappropriate, such as the low burst size for the models with only the adsorption rate linked to bacterial growth (12 [10 to 14] and 10 [8 to 12]) or the high latent period for the models with both adsorption rate and burst size linked to bacterial growth (0.93 [0.86 to 0.99] and 0.88 [0.79 to 0.96]) (Table 1).

We used our best-fitting model to reproduce our *in vitro* data (Fig. 2) and uncover the underlying phage-bacteria dynamics. Due to the link between phage burst size and bacterial growth, burst size decreases as bacteria reach carrying capacity after 8 h (Fig. 6a and b). This is reflected in the relative change in phage numbers, which tends toward 0 after 8 h (Fig. 6b). After this point, phage incidence remains stable for the 10^3^ and 10^4^ PFU/mL data set but starts increasing again significantly after 20 h for the 10^5^ PFU/mL data set as bacterial numbers start decreasing due to phage predation, allowing burst size to increase again (Fig. 6a to c).

We estimate that for every 10^8^ new lytic phage released during burst, there was approximately one transducing phage carrying an antibiotic resistance gene (Table 1 and Fig. 6c). Note that new DRP can be generated either by transduction or by replication of already existing DRP. Using the model, we found that DRP were always predominantly generated by transduction rather than by growth (Fig. 6d). This is because DRP only appear after 2 to 4 h, while after 4 h bacterial growth rate starts decreasing as the total bacteria population approaches carrying capacity (Fig. 6b and d).

## DISCUSSION

### Results in context.

We observed rapid *in vitro* horizontal gene transfer of antimicrobial resistance (AMR) by generalized transduction in Staphylococcus aureus alongside equilibria in phage and bacterial numbers, which varied depending on the starting number of phage. The most accurate mathematical model to replicate phage-bacteria dynamics, including generalized transduction, represented phage predation as a frequency-dependent interaction and linked phage burst size to bacterial growth. To the best of our knowledge, these two elements have both been suggested previously (17, 18, 36) but never combined.

Density-dependent models have been compared to data at less fine time scales (e.g., daily time points) or over smaller time periods (e.g., less than 8 h), where they were able to reproduce *in vitro* values from experiments in chemostats and have been helpful to improve our basic understanding of phage-bacteria dynamics (14–16). However, here we show that this type of interaction is not able to reproduce finer hourly dynamics and does not perform consistently when varying concentrations of starting phage and bacteria. Using this, alongside a critique of the mathematical implications of this process, we argue that density dependence is not a biologically accurate representation of phage predation, as it fails to reproduce these dynamics at large or small numbers of phage and bacteria, which would correspond to scenarios potentially seen during phage therapy.

Our work adds to the growing body of evidence that phage predation depends on bacterial growth (14, 17–23). This has implications for antibiotic-phage combination therapy, as it suggests that bacteriostatic antibiotics, which prevent bacterial growth, could reduce phage predation. This effect has been seen previously in S. aureus (40). In the environment, including in persistent infections, bacteria spend most of their time in stationary phase (41). This suggests that bacteria and phage can coexist for prolonged periods of time in a broad range of settings without the phage systematically eradicating the bacteria. Under such conditions, phage may mediate horizontal gene transfer by transduction between bacteria at relatively low levels but for prolonged periods of time. This may be particularly relevant for S. aureus, since approximately 20% of humans are colonized asymptomatically by this bacterium at any given time (42), and at least 50% of these carriers may also carry phage capable of generalized transduction (43), suggesting a constant background evolution rate for S. aureus in the human population. Combined with environmental exposure to antibiotics, which acts as a selective pressure, this may contribute to the risk of multidrug-resistant bacterial evolution.

### Strengths and limitations.

Our experimental design is both a strength and a limitation of our study. Since we jointly designed the experiments and models, we are confident that we have included in our mathematical model all the organisms and interactions present *in vitro*. We are therefore confident in the conclusions on model structure, which is generalizable to other systems. Frequency-dependent predation is biologically plausible for lytic phage in general, a link between phage predation and bacterial growth has been seen in other systems (14, 17–23), and our model includes the relevant biological characteristics of generalized transduction (2, 3), requiring a transducing phage to first be generated before the transfer of the AMR gene to another bacterium can occur. In addition, our equations for phage-bacteria interaction can be directly applied to systems containing more strains of phage and bacteria than in our study.

However, the use of such a specific experimental system with two bacterial strains of the same genetic background and one phage limits the generalizability of our parameter values, as these will likely vary for different bacteria and phage. Growth conditions will likely also differ between the *in vitro* environment studied here and *in vivo* conditions. Here, our model assumes that phage do not decay, that bacteria do not become resistant to phage, and that they can grow indefinitely as they are observed in a rich medium for 24 h only, but over longer periods of time it may be necessary to revisit these assumptions (44). The role of the immune system may have to be considered *in vivo*, as this could impact the numbers of phage and bacteria (45, 46), and our model could be extended to include this. We assumed that the proportion of transducing phage created was independent of the gene being transduced [*erm*(B) on the bacterial chromosome or *tet*(K) on a plasmid]. This was supported by preliminary work (not shown) but should be further investigated to improve our understanding of the factors that can facilitate or prevent transduction of different genes. Finally, our model does not include lysogeny and specialized transduction and would therefore need to be extended with additional compartments for lysogenic bacteria to represent these dynamics. To answer all of these questions, future work should investigate both phage predation and transduction dynamics over longer time periods with different strains of bacteria and phage.

All our models captured certain aspects of the trends seen *in vitro* but also underestimated phage numbers between 5 and 7 h by up to 20 times. This is likely a consequence of our experimental design. To count lytic phage, we centrifuged and filtered the coculture to remove bacteria. This could have caused the premature burst of some phage-infected bacteria, artificially increasing the numbers of phage we then counted (47). Since the period between 5 and 7 h is when phage infections are highest (Fig. 6b), this is why we would see such a large discrepancy at this stage. We also note that the models with only phage burst size linked to bacterial growth underestimated the number of double-resistant progeny (DRP). This small difference (up to 10 CFU/mL) is likely due to our choice of using a deterministic model. This type of model is useful for our purpose of fitting to *in vitro* data and analyzing the underlying dynamics here but mathematically allows for fractions of bacteria to exist instead of just whole numbers. Future analyses using a stochastic model would better capture random effects, which can have an important impact at low numbers.

Multiplicity of infection (MOI; starting ratio of phage to bacteria) is a commonly used metric to present results of experiments with these organisms (32). With a starting concentration of 10^4^ bacteria per mL, we were able to fit our model to the dynamics for two MOI (0.1 and 10) and replicate those of a third (1). However, when trying to use the same model for these same three MOI but with a starting bacterial concentration at 10^6^, we found differences between our model and values seen after 24 h. This indicates that MOI is not appropriate to summarize all the complexity of the underlying phage-bacteria dynamics. Future experimental studies should express their results as a function of their starting concentration of phage and bacteria, not just MOI.

In any case, the failure of our model to replicate 24 h values with different starting bacterial concentrations shows that while we have reduced the model structure uncertainty, we are still not fully capturing the phage-bacteria interaction. Currently, our model predicts that, for a starting concentration of 10^6^ bacteria, a starting concentration of 10^5^ phage or more will be enough to cause a decrease in bacterial numbers after 24 h, while our data show that the starting concentration of phage must be higher than 10^6^ for this to happen. *In vitro*, it is likely that slower bacterial growth simultaneously affects the phage adsorption rate, latent period, and burst size, each to various extents (14, 17–23). This would explain why we would need a higher starting concentration of phage for a higher starting concentration of bacteria to exert a strong enough predation pressure before bacteria reach stationary phase, causing a reduction in phage predation. However, here we have only made the first step in this process, having linked the burst size linearly to the bacterial growth rate, instead of trying to link different phage predation parameters to bacterial growth using different functions. These complexities need to be explored further, supported by *in vitro* work measuring phage predation parameters at various time points. In S. aureus, wall teichoic acid (WTA) is the phage receptor (48, 49). Lack of WTA glycosylation has been shown to induce phage resistance (50), and changes in WTA structure at different growth phases may be possible, since one of the genes involved in its synthesis is repressed by a quorum-sensing system (51). However, to the best of our knowledge this has not yet been investigated.

### Implications.

Despite being recognized as a major mechanism of horizontal gene transfer, thus far there have been limited mathematical modeling studies on the dynamics of transduction of AMR (12). Using our model, we are able to estimate numbers of transducing phage that we cannot count *in vitro* and see that approximately 1 generalized transducing phage is generated per 10^8^ lytic phage, consistent with previous estimates (52, 53). Here, we show that this number, which may seem insignificant, is enough to consistently lead to the successful horizontal gene transfer of AMR, resulting in DRP after only 7 h from phage addition, substantially less than the usual duration of antibiotic treatment. We also show that transduction is the dominant mechanism to create new DRP throughout the experiment rather than growth of existing DRP. This echoes the conclusions of previously published work on the importance of transduction, including *in vivo* experiments and with other Staphylococcus species (4, 5, 29, 54).

Our findings suggest that transduction is currently underemphasized in the exploration of phage-bacteria dynamics. Future studies on this topic should not assume that transduction can be dismissed by default but instead investigate whether it is relevant in their system. This requires further *in vitro* and *in vivo* monitoring to identify scenarios where transduction plays a significant role in the transfer of AMR genes, likely depending on the environment and characteristics of the bacteria and phage present. This will require new experimental designs, since counting phage numbers can be difficult, notably with clinical strains of bacteria. This should also be investigated in the presence of antibiotics, where the importance of selection enters, increasing the fitness of the small numbers of DRP generated by transduction.

Our results confirm that generalized transduction can consistently lead to the spread of AMR genes, yet to the best of our knowledge there have not been any attempts to evaluate the potential consequences of this process during phage therapy. Unlike specialized transduction, likely not relevant in the context of phage therapy as temperate phage would not be used for this purpose, generalized transduction is by definition a mistake during the lytic cycle and therefore is difficult to prevent (8, 9). As phage therapy is generally administered alongside antibiotics (55) and we know that patients can be colonized and infected with strains carrying different resistance genes (42), a potential risk is for multidrug-resistant strains to be generated by transduction and then selected for by these antibiotics. These new strains could in turn be transmitted to other individuals or gain resistance to phage infection, which would lead to a worse treatment outcome for the patient. Echoing recommendations from previous reviews (10–12), we suggest that future studies of phage therapy should acknowledge the risk of generalized transduction and evaluate the impact of this on *in vivo* bacterial evolution during therapy.

### Conclusions.

The joint dynamics of phage predation and transduction lead to complex interactions with bacteria. These dynamics must be clarified to correctly evaluate the extent to which phage contribute to the global spread of AMR. We must also understand these dynamics in the context of phage therapy, as transduction may lead to worse health outcomes in patients if phage contribute to spreading AMR instead of overcoming it. Current modeling research that ignores transduction may underestimate AMR development in various systems. Interdisciplinary work will be essential to answer these urgent public health questions in the near future.

## MATERIALS AND METHODS

All analyses were conducted using the statistical software R (56). The underlying code and data are available in a GitHub repository: https://github.com/qleclerc/mrsa_phage_dynamics.

### Experimental methods. (i) Strains and phage used.

The Staphylococcus aureus parent strains used for our transduction experiment were obtained from the Nebraska Transposon Mutant Library (35). These were strain NE327, carrying the *erm*(B) gene encoding erythromycin resistance and knocking out the ϕ3 integrase gene, and strain NE201KT7, a modified NE201 strain with a kanamycin resistance cassette instead of the *erm*(B) gene knocking out the ϕ2 integrase gene and a pT181 plasmid carrying the *tet*(K) gene encoding tetracycline resistance (57). Growing these strains together under identical conditions as those for our coculture below, but without the addition of exogenous phage, does not lead to detectable horizontal gene transfer (HGT; data not shown). To enable HGT, exogenous 80α phage was used, a well-characterized temperate phage of S. aureus capable of generalized transduction (33). To count lytic phage, S. aureus strain RN4220 was used, a restriction-deficient strain highly susceptible to phage infection (58).

### (ii) Transduction coculture protocol.

Precultures of NE327 and NE201KT7 were prepared separately in 50 mL conical tubes with 10 mL of brain heart infusion broth (BHIB; Sigma, United Kingdom) and incubated overnight in a shaking water bath (37°C, 90 rpm). The optical densities of the precultures were checked at 625 nm the next day to confirm growth. The precultures were diluted in phosphate-buffered saline (PBS) and added to a glass bottle of fresh BHIB to reach the desired starting concentration in CFU per mL (CFU/mL) for each strain, forming a master mix for the coculture. CaCl_2_ was added at a concentration of 10 mM to the master mix. Phage 80α stock was diluted in phage buffer (50 mM Tris-HCl, pH 7.8, 1 mM MgSO_4_, 4 mM CaCl_2_, and 1 g/liter gelatin; Sigma-Aldrich) and added to the master mix to reach the desired starting concentration in PFU per mL; 10 50 mL conical tubes were prepared (one coculture tube for each time point, from 0 to 8 h and 16 to 24 h), each with 10 mL from the master mix. Each coculture tube was then incubated in a shaking water bath (37°C, 90 rpm) for the corresponding duration.

Bacterial counts for each time point were obtained by diluting the coculture in PBS before plating 50 μL on selective agar, either plain brain heart infusion agar (BHIA; Sigma, United Kingdom), BHIA with erythromycin (Sigma, United Kingdom) at 10 mg/liter, BHIA with tetracycline (Sigma, United Kingdom) at 5 mg/liter, or BHIA with both erythromycin and tetracycline (10 mg/liter and 5 mg/liter). Note we plated 500 μL instead of 50 on the plates with both antibiotics to increase the sensitivity of the assay. This allowed distinction between each parent strain, resistant to either erythromycin or tetracycline, and the DRP generated by transduction. Plates were then incubated at 37°C for 24 h or 48 h for plates containing both antibiotics. Colonies were counted on the plates to derive the CFU/mL in the coculture for that time point.

Lytic phage counts for each time point were obtained using the agar overlay technique (59). Briefly, the coculture was centrifuged at 4,000 rpm for 15 min, filtered twice with 10 μm filters, and diluted in nutrient broth no. 2 (NB2; ThermoFisher Scientific, United Kingdom); 15 mL conical tubes were prepared with 300 μL of RN4220 grown overnight in NB2 and CaCl_2_ at a concentration of 10 mM; 200 μL of diluted phage was added, and the tubes were left to rest on the bench for 30 min. The contents of the tubes were then mixed with 7 mL of phage top agar and poured on phage agar plates. Phage agar was prepared using NB2, supplemented with agar (Sigma, United Kingdom) at 3.5 g/liter for top agar and 7 g/liter for plates. The plates were incubated overnight at 37°C. Clear spots in the bacterial lawn were counted to derive the PFU/mL in the coculture for that time point.

### (iii) Relative fitness.

Relative fitness was calculated using data from cocultures of NE327, NE201KT7, and DRP in the absence of phage. For each pair of strains, we estimated relative fitness, *W*, using equation 32.

(32) $$W\;=\;\frac{\text{ln}[\frac{S1(24)}{S1(0)}]}{\text{ln}[\frac{S2(24)}{S2(0)}]}$$

Where *S*1(*t*) and *S*2(*t*) represent the number of bacteria (in CFU/mL) from the chosen strains 1 and 2 at times t = 0 or 24 h.

### (iv) PCR protocols.

To confirm that DRP contained both the *erm*(B) and *tet*(K) genes, primers ermBF (5′-CGTAACTGCCATTGAAATAGACC-3′), ermBR (5′-AGCAAACTCGTATTCCACGA-3′), tetKF (5′-ATCTGCTGCATTCCCTTCAC-3′), and tetKR (5′-GCAAACTCATTCCAGAAGCA-3′) were used. Strains NE327 [only containing *erm*(B)] and NE201KT7 [only containing *tet*(K)] were used as positive and negative controls.

To confirm that 80α lysogeny did not occur in our coculture, we applied a previously published method (33) and used a combination of four primers: SaRpmF (5′-GACTGAATGCCCAAACTGTG-3′) in the S. aureus *rpmF* gene, SMT178 (5′-GGCTGGGAATTAATGGAAGATG-3′) in the 80α integrase, SaSirH (5′-TTAAGTAGCATCGTTGCATTCG-3′) in the S. aureus *sirH* gene, and SMT179 (5′-GAGTCCTGTTTGCGAATTAGG-3′) in the 80α ORF73 region. SaRpmF and SMT178 were used to amplify the left prophage junction (*attL*), SaSirH and SMT179 to amplify the right junction (*attR*), and SaRpmF and SaSirH to amplify the bacterial insertion site (*attB*). RN4220 was used as a negative control for lysogeny, and JP8488, an RN4220 strain lysogenic for 80α, was used as a positive control (obtained from José Penadés and Nuria Quiles, Imperial College London).

All PCRs were conducted using OneTaq hot start quick-load 2× master mix by following the manufacturer’s protocol. Tested samples were homogenized in 20 μl nuclease-free water, except for samples used to test for lysogeny that were generated by DNA extraction and therefore already suspended in nuclease-free water (see below); 1.5 μl of each suspension was used as the template for a total reaction volume of 25 μl.

### (v) DNA extraction protocol.

To prepare samples for PCR to detect lysogeny, we extracted DNA from a 1 mL sample of our NE327, NE210KT7, and 80α coculture after 24 h (approximately 10^9^ bacteria) using the bacterial genomic DNA purification kit PurElute (Edge Biosystems), supplemented with 2.5 μl of lysostaphin (10 mg/mL; Sigma-Aldrich) (43).

The final DNA suspension was in 50 μl of nuclease-free water, and we used 1.5 μl of this suspension as a template for the PCR and conducted three experimental replicates, which is equivalent to saying that we tested DNA from approximately 9 × 10^7^ bacteria (10^9^ × [1.5/50] × 3 = 9 × 10^7^). Using a binomial probability density function and assuming a 100% PCR specificity, we estimate that the probability for a false-negative result (i.e., that the PCR results are negative yet the true number of lysogenic bacteria is greater than 0) exceeds 5% only if the frequency of lysogenic bacteria in our sample is lower than 3.3 × 10^−8^. We therefore consider that the detection limit of our protocol is a frequency of 3.3 × 10^−8^ lysogenic per nonlysogenic bacteria after 24 h of our coculture. This means that, in our system, we would be able to detect lysogenic bacteria if there were more than (3.3 × 10^−8^ × 10^9^ = 33) 33 lysogenic bacteria in 1 mL of our coculture after 24 h.

### Mathematical modeling methods. (i) General model structure.

We designed a deterministic, compartmental model to replicate our experimental conditions. We included 6 populations: B_E_ (corresponding to erythromycin-resistant NE327), B_T_ (tetracycline-resistant NE201KT7), B_ET_ (DRP), P_L_ (lytic phage), P_E_ [phage transducing *erm*(B)], and P_T_ [phage transducing *tet*(K)]. Their interactions are represented in Fig. 2.

Bacteria from each strain, θ (θ ∈ {E, T, ET}), can multiply at each time step, *t*, by following logistic growth at rate μ<συβ>θ</συβ>, with a maximum value, μ_maxθ_, which declines as the total bacteria population, *N* (= B_E_ + B_T_ + B_ET_), approaches carrying capacity, *N*_max_.

(33) $$\mu_{\theta }=\;\mu_{{\text{max}}_{\text{θ}}}\times (1-\frac{N}{N_{\text{max}}})$$

At each time step, *t*, a proportion, λ, of lytic phage (P_L_) infect a number of bacteria (φ_L_), replicate, and burst out from the bacteria with a burst size, δ + 1, after a latent period, τ. During phage replication, a proportion, α, of new phage are transducing phage. The nature of the transducing phage (P_E_ or P_T_) depends on the bacteria being infected (e.g., B_E_ bacteria can only lead to P_E_ phage). A proportion, λ, of these transducing phage (P_E_ or P_T_) infect a number of bacteria (φ_E_ or φ_T_). If they successfully infect a bacterium carrying the other resistance gene (e.g., P_E_ phage infecting a B_T_ bacterium), this creates DRP (B_ET_). The complete model equations can be found below.

(34) $$\frac{dB_{E}}{\mathrm{dt}}=\mu_{E}\times (B_{E}-\text{ω}\times \left(\left(\varphi_{L}+\varphi_{T}\right)\times \frac{B_{E}}{N}\right))-\left(\varphi_{L}+\varphi_{T}\right)\times \frac{B_{E}}{N}$$

$$\left\{\text{Change in }B_{E}=\text{ growth of }B_{E}–\text{ infections by }P_{L}–\text{ infections by }P_{T}\right\}$$

(35) $$\frac{dB_{T}}{\mathrm{dt}}=\mu_{T}\times (B_{T}-\text{ω}\times \left(\left(\varphi_{L}+\varphi_{E}\right)\times \frac{B_{T}}{N}\right))-\left(\varphi_{L}+\varphi_{E}\right)\times \frac{B_{T}}{N}$$

$$\left\{\text{Change in }B_{T}=\text{ growth of }B_{T}–\text{ infections by }P_{L}–\text{ infections by }P_{E}\right\}$$

(36) $$\frac{dB_{\text{ET}}}{\mathrm{dt}}=\mu_{\text{ET}}\times (B_{\text{ET}}-\text{ω}\times \left(\varphi_{L}\times \frac{B_{\text{ET}}}{N}\right))-\varphi_{L}\times \frac{B_{\text{ET}}}{N}+\varphi_{E}\times \frac{B_{T}}{N}+\varphi_{T}\times \frac{B_{E}}{N}$$

$$\left\{\text{Change in }B_{\text{ET}}=\text{ growth of }B_{\text{ET}}–\text{ infections by }P_{L}+\text{ infections of }B_{T}\text{ by }P_{E}+\text{ infections of }B_{E}\text{ by }P_{T}\right\}$$

(37) $$\frac{dP_{L}}{\mathrm{dt}}=\varphi_{L}(t-\text{τ})\times \delta \times \left(1-\text{α}\times \frac{B_{E}+B_{T}+2\times B_{\text{ET}}}{N}\right)-\lambda \times P_{L}$$

$$\left\{\text{Change in }P_{L}=\text{ new }P_{L}\text{ phage }–\;P_{L}\text{ phage infecting bacteria}\right\}$$

(38) $$\frac{dP_{E}}{\mathrm{dt}}=\varphi_{L}(t-\text{τ})\times \delta \times \text{α}\times \frac{B_{E}+B_{\mathrm{ET}}}{N}-\lambda \times P_{E}$$

$$\left\{\text{Change in }P_{E}=\text{ new }P_{E}\text{ phage }–\;P_{E}\text{ phage infecting bacteria}\right\}$$

(39) $$\frac{dP_{T}}{\mathrm{dt}}=\varphi_{L}(t-\text{τ})\times \delta \times \text{α}\times \frac{B_{T}+B_{\text{ET}}}{N}-\lambda \times P_{T}$$

$$\left\{\text{Change in }P_{T}=\text{ new }P_{T}\text{ phage }–\;P_{T}\text{ phage infecting bacteria}\right\}$$

Some parameters (τ, α, ω) are constant, while others (μ_E_, μ_T_, μ_ET_, β, φ_L_, φ_E_, φ_T_, δ) can change at each time step and depending on the specified interaction mechanism. Note that ω is a special parameter equal to 0 if the model is density-dependent or 1 if it is frequency-dependent.

### (ii) Density-dependent interaction.

Over one time step, both the number of phage infecting bacteria and the number of bacteria infected by phage are equal to the product of the number of phage, bacteria, and phage adsorption rate. In our equations for density dependence, given the phage adsorption rate, β, the proportion, λ, of phage that infect any bacteria is

(40) λ=β×N

and the number of bacteria infected by a phage θ (θ ∈ {L, E, T}) is

(41) $$\varphi_{\theta }=\lambda \times P_{\theta }$$

Note that the parameter ω is set to 0 in this case.

### (iii) Frequency-dependent interaction.

Using this interaction prevents the number of phage-infecting bacteria over one time step from being higher than the total number of phage in the system (and the number of bacteria being infected one time step being higher than the total number of bacteria in the system). Equations 40 and 41 then become

(42) λ=[1−exp (−β ×N)]

(43) $$\varphi_{\theta }=\left(1-\text{exp }\left(-\lambda \times \frac{P_{\theta }}{N}\right)\right)\;\times N$$

With the frequency-dependent interaction, we set the parameter ω to 1. This ensures that, over one time step and for any bacterium, phage infection and bacterial replication are mutually exclusive events. Without this modification, phage infections would not be able to reduce bacterial population size due to mathematical constraints. Equation 44 shows a simplified frequency-dependent model for a single bacterial strain, *B*, without any correction term.

(44) $$\frac{\mathrm{dB}}{\mathrm{dt}}=\mu_{\text{max}}\times (1-\frac{B}{B_{\text{max}}})\times B-\varphi_{\theta }\times B$$

According to equation 44, the change in bacterial numbers depends on the relative values of bacterial growth and bacterial death due to phage predation, expressed in equation 45.

(45) $${\text{μ}}_{\text{max}}\times (1-\frac{B}{B_{\text{max}}})-\text{φ}$$

The necessity for a correction term on the left side of equation 44 arises from the maximum values of μ_max_ and φ_θ_. As can be deduced from equation 43, the maximum possible value for φ_θ_ is 1. According to our fitted parameter values, the maximum value for μ_max_ in our model is approximately 1.5, and the carrying capacity, *B*_max_, is approximately 2.8 × 10^9^. Substituting these into equation 45 leads to equation 46.

(46) $$1.5\times (1-\frac{B}{2.8\times 10^{9}})-1$$

To have a decline in the bacterial population, we therefore must satisfy the condition stated in equation 47.

(47) $$1.5\;\times (1-\frac{B}{2.8\times 10^{9}})<1$$

This can be rearranged into equation 48 by dividing by 1.5.

(48) $$1-\frac{B}{2.8\times 10^{9}}<\frac{1}{1.5}$$

Finally, by subtracting 1 and multiplying by −2.8 × 10^9^, we obtain equation 49.

(49) $$B>-(\frac{1}{1.5}-1)\times 2.8\times 10^{9}$$

Solving equation 49 leads to the condition that the number of bacteria must be greater than 9.3 × 10^8^ for bacterial growth to be low enough that bacterial numbers decrease. In other words, it is impossible for the effect of phage predation to decrease bacterial numbers below 9.3 × 10^8^. We overcome this by applying a correction term to Equation S1, leading to equation 50.

(50) $$\frac{\mathrm{dB}}{\mathrm{dt}}={\text{μ}}_{\text{max}}\times (1-\frac{B}{B_{\text{max}}})\times (B-\varphi_{\theta }\times B)-\varphi_{\theta }\times B$$

This is equivalent to saying that bacteria infected by phage cannot replicate and, hence, is more biologically realistic.

### (iv) Link between bacterial growth and phage predation.

We consider that reduced bacterial growth can lead to decreased phage predation through reduced adsorption (β) and/or burst size (δ). Equations 51 and 52 allow these parameters to decrease as bacterial growth decreases, using the same principle of logistic growth as that seen in equation 33.

(51) $$\beta ={\text{β}}_{\text{max}}\times \left(1-\frac{N}{N_{\text{max}}}\right)$$

(52) $$\delta ={\text{δ}}_{\text{max}}\times \left(1-\frac{N}{N_{\text{max}}}\right)$$

If we do not link these parameters to bacterial growth, we assign them their maximum values.

(53) $$\beta ={\text{β}}_{\text{max}}$$

(54) $$\delta ={\text{δ}}_{\text{max}}$$

### (v) Model fitting.

We fit our model to the *in vitro* data using the Markov chain Monte Carlo Metropolis–Hastings algorithm. For every iteration, this algorithm slightly changes the parameter values, runs the model, assesses the resulting model fit to the data, and accepts or rejects these new parameter values based on whether the model fit is better or worse than that with the previous set of values. We run the algorithm with two chains, and once convergence has been reached (determined using the Gelman-Rubin diagnostic, once the multivariate potential scale reduction factor is less than 1.2 [60]), we generate 50,000 samples from the posterior distributions for each parameter.

In a first instance, we used our growth coculture data, where phage are absent, to calibrate the bacterial growth rate parameters, μ_maxθ_, for each bacteria strain, θ (θ ∈ {E, T, ET}), and the carrying capacity, *N*_max_, using a simple logistic growth model (equation 55). All other parameters related to phage predation were set to 0.

(55) $$\frac{dB_{\theta }}{\mathrm{dt}}=\mu_{{\text{max}}_{\text{θ}}}\times B_{\theta }\times \left(1-\frac{B_{\theta }}{N_{\text{max}}}\right)$$

The phage predation parameters (τ, α, β_max_, δ_max_) were jointly estimated by fitting to the phage and double-resistant bacterial numbers from the transduction coculture data. We fitted to the transduction coculture data sets with starting phage concentrations of 10^3^ and 10^5^ PFU/mL and tested whether the estimated parameters could reproduce the dynamics seen with the starting phage concentration of 10^4^ PFU/mL. Convergence and posterior distribution plots for our best-fitting model are shown in Fig. S6 in the supplemental material. Fitting was performed by evaluating the log likelihood of each *in vitro* data point being observed in a Poisson distribution, with the corresponding model data point as a mean.

10.1128/msystems.00135-22.6 FIG S6 Convergence and posterior distributions for the best-fitting model (with a frequency-dependent interaction and a link between phage burst size and bacterial growth). (a to d) Parameter convergence plots. Fitting was performed using two chains (black and red). (e to h) Posterior distributions. The prior distributions for the burst size and latent period are shown in blue. The prior distributions for other parameters were uninformative and are not shown. Download FIG S6, TIF file, 1.9 MB. Copyright © 2022 Leclerc et al. 2022 Leclerc et al. https://creativecommons.org/licenses/by/4.0/ This content is distributed under the terms of the Creative Commons Attribution 4.0 International license.

To mirror our experimental sampling variation, *in vitro* data points were scaled down to be between 1 and 100 before fitting, with the same correction applied to the corresponding model-predicted value for the same time point. For example, if at 1 h there are 1.4 × 10^4^ phage *in vitro*, this is scaled down to 14, and if the corresponding model value is 5.3 × 10^6^, this is scaled down by the same magnitude (i.e., 10^3^), resulting in a value of 5,300.

Previous research estimated that the latent period for 80α in S. aureus was approximately 40 min (0.67 h) and that the burst size was approximately 40 phage per bacterium (39). Since this study did not provide error values for these point estimates, we assumed the standard deviation and chose the following informative priors for these parameters: τ ∼ Normal(0.67, 0.07) (95% confidence interval, 0.53 to 0.81) and δ_max_ ∼ Normal(40, 7) (95% confidence interval, 54 to 26). Due to a lack of available data, we used uninformative priors for the remaining parameters: α ∼ Uniform(0, 1) and β_max_ ∼ Uniform(0, 1).
