# Correction for Leclerc et al., “Growth-Dependent Predation and Generalized Transduction of Antimicrobial Resistance by Bacteriophage”

**DOI:** 10.1128/msystems.00974-22

**Published:** 2022-12-12

**Authors:** Quentin J. Leclerc, Jacob Wildfire, Arya Gupta, Jodi A. Lindsay, Gwenan M. Knight

## AUTHOR CORRECTION

Volume 7, no. 2, e00135-22, 2022, https://doi.org/10.1128/msystems.00135-22. Following discussions with colleagues and peer reviewers on further work extending the model introduced in the manuscript, we have identified a technical issue in the way we mathematically phrased the phage-bacterium interaction. This has been corrected with an improved equation which restricts the maximum rate of phage predation at higher phage concentrations, exactly as our original frequency-dependent approach did, but with greater biological and mathematical clarity. Refitting the model with this improved “saturated phage predation” term does not substantially change our parameter values, figures, results, or conclusions.

All mentions of “frequency-dependent” phage predation should be replaced by “saturated” phage predation, and all mentions of “density-dependent” phage predation should be replaced by “linear” phage predation.

Page 6: The two equations used for the frequency-dependent interaction (equations 2 and 3) should be replaced by this single improved equation for saturated predation:

$$B\times P\times \frac{\beta }{\left(1+\frac{P}{P_{\text{50}}}\right)}$$

where *P*_50_ corresponds to the phage concentration at half-saturation, i.e., where the adsorption rate is equal to half the maximum.

Page 11: Figure 5 should appear as shown below, with the results from fitting the model with the improved “saturated phage predation” term.[Fig fig5]

**Figure fig5:**
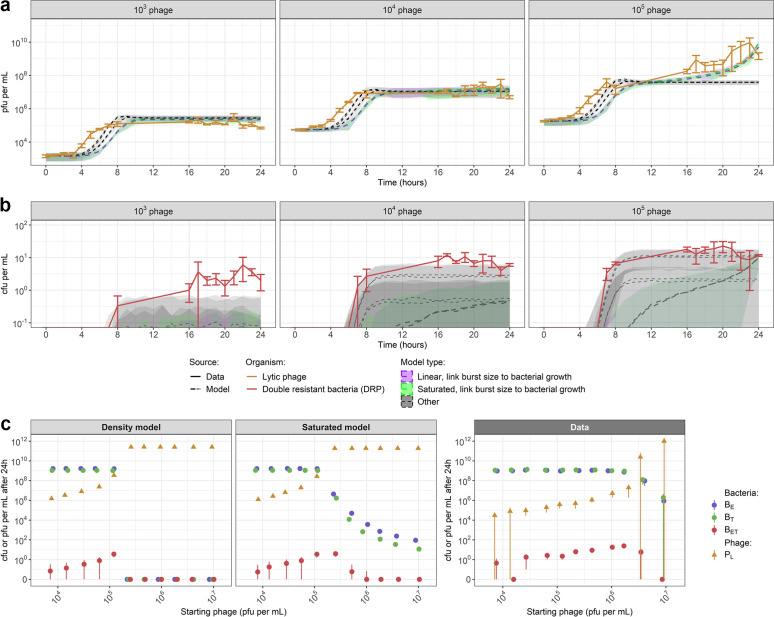


Page 12: Table 1 should appear as shown below.

**Table tab1:** 

Interaction type	Adsorption rate linked to growth	Burst size linked to growth	Adsorption rate β (phage^−1^ bacteria^−1^ h^−1^)	Burst size δ (phage)	Transducing phage proportion α (proportion of burst size)	Phage latent period τ (h)	Phage concn at half-saturation *P*_50_ (phage)	DIC
Linear	Yes	No	4.5 × 10^−9^ (4.1 × 10^−9^; 5.0 × 10^−9^)	12 (10; 14)	3.1 × 10^−8^ (1.5 × 10^−8^; 5.8 × 10^−8^)	0.64 (0.55; 0.73)	N/A	610
	No	Yes	1.6 × 10^−10^ (1.5 × 10^−10^; 1.7 × 10^−10^)	79 (72; 86)	1.4 × 10^−8^ (1.1 × 10^−8^; 1.7 × 10^−8^)	0.65 (0.62; 0.69)	N/A	63
	Yes	Yes	4.3 × 10^−9^ (3.9 × 10^−9^; 4.6 × 10^−9^)	43 (37; 49)	1.2 × 10^−8^ (6.4 × 10^−9^; 2.3 × 10^−8^)	0.93 (0.86; 0.99)	N/A	298
Saturated	Yes	No	3.3 × 10^−9^ (1.8 × 10^−9^; 5.6 × 10^−9^)	14 (11; 21)	2.5 × 10^−7^ (1.2 × 10^−7^; 5.5 × 10^−7^)	0.67 (0.60; 0.78)	5.1 × 10^10^ (2.8 × 10^9^; 9.7 × 10^10^)	631
	No	Yes	2.3 × 10^−10^ (2.1 × 10^−10^; 2.7 × 10^−10^)	50 (43; 54)	1.2 × 10^−8^ (1.1 × 10^−9^; 1.3 × 10^−8^)	0.60 (0.60; 0.61)	1.2 × 10^10^ (1.0 × 10^10^; 1.3 × 10^10^)	0
	Yes	Yes	2.6 × 10^−9^ (1.9 × 10^−9^; 3.4 × 10^−9^)	36 (28; 43)	1.4 × 10^−7^ (9.21 × 10^−8^; 2.2 × 10^−7^)	0.75 (0.63; 0.80)	5.1 × 10^10^ (3.6 × 10^9^; 9.8 × 10^10^)	385

Pages 18 and 19: Equations 34 to 39 should read as follows:[Table tab1]

$$\frac{dB_{E}}{\mathrm{dt}}=\mu_{E}\times B_{E}-B_{E}\times F(P_{L})-\;B_{E}\times F(P_{T})$$

$$\frac{dB_{T}}{\mathrm{dt}}=\mu_{T}\times B_{T}-B_{T}\times F(P_{L})-B_{T}\times F(P_{E})$$

$$\frac{dB_{\mathrm{ET}}}{\mathrm{dt}}=\mu_{\mathrm{ET}}\times B_{\mathrm{ET}}-B_{\mathrm{ET}}\times F(P_{L})+B_{T}\times F(P_{E})+B_{E}\times F(P_{T})$$

$$\frac{dP_{L}}{\mathrm{dt}}=\left[\left(B_{E}+B_{T}\right)\times F\left(P_{L}\right)\right](t-\text{τ})\times \delta \times \left(1-\text{α}\right)+[B_{\mathrm{ET}}\times F\left(P_{L}\right)]\left(t-\text{τ}\right)\times \delta \times \left(1-2\text{*α}\right)-N\times F(P_{L})$$

$$\frac{dP_{E}}{\mathrm{dt}}=[\left(B_{E}+B_{\mathrm{ET}}\right)\times F\left(P_{L}\right)](t-\text{τ})\times \delta \times \text{α}-N\times F(P_{E})$$

$$\frac{dP_{T}}{\mathrm{dt}}=[\left(B_{T}+B_{\mathrm{ET}}\right)\times F\left(P_{L}\right)](t-\text{τ})\times \delta \times \text{α}-N\times F(P_{T})$$

Page 19: The two equations used for the density-dependent interaction (equations 40 and 41) should be replaced by this single equation, to be consistent with the model equations above:

$$F\left(P_{\theta }\right)=P_{\theta }\times \beta$$

Page 19: The two equations used for the frequency-dependent interaction (equations 42 and 43) should be replaced by this single improved equation for saturated predation:

$$F\left(P_{\theta }\right)=P_{\theta }\times \frac{\beta }{\left(1+\frac{P_{\theta }}{P_{\text{50}}}\right)}$$

Page 22: Reference 36 should read as follows: Roach DR, Leung CY, Henry M, Morello E, Singh D, Di Santo JP, Weitz J S, Debarbieux L. 2017. Synergy between the host immune system and bacteriophage is essential for successful phage therapy against an acute respiratory pathogen. Cell Host Microbe 22:38–47.e4.

