# Supplementary figures and images for: Growth-Dependent Predation and Generalized Transduction of Antimicrobial Resistance by Bacteriophage
Source: mSystems. 2022 Mar 21;7(2):e00135-22. doi: 10.1128/msystems.00135-22 (PMC9040582; doi:10.1128/msystems.00135-22)

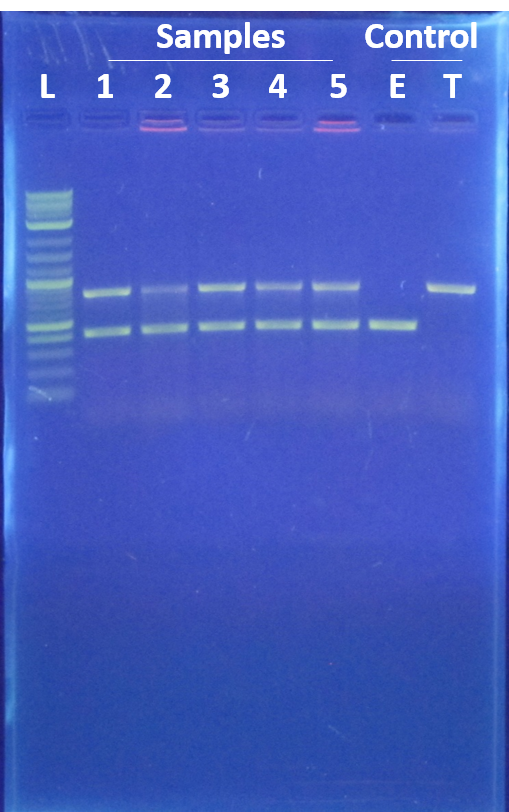

Supplement: FIG S1 [file msystems.00135-22-sf001.tif]

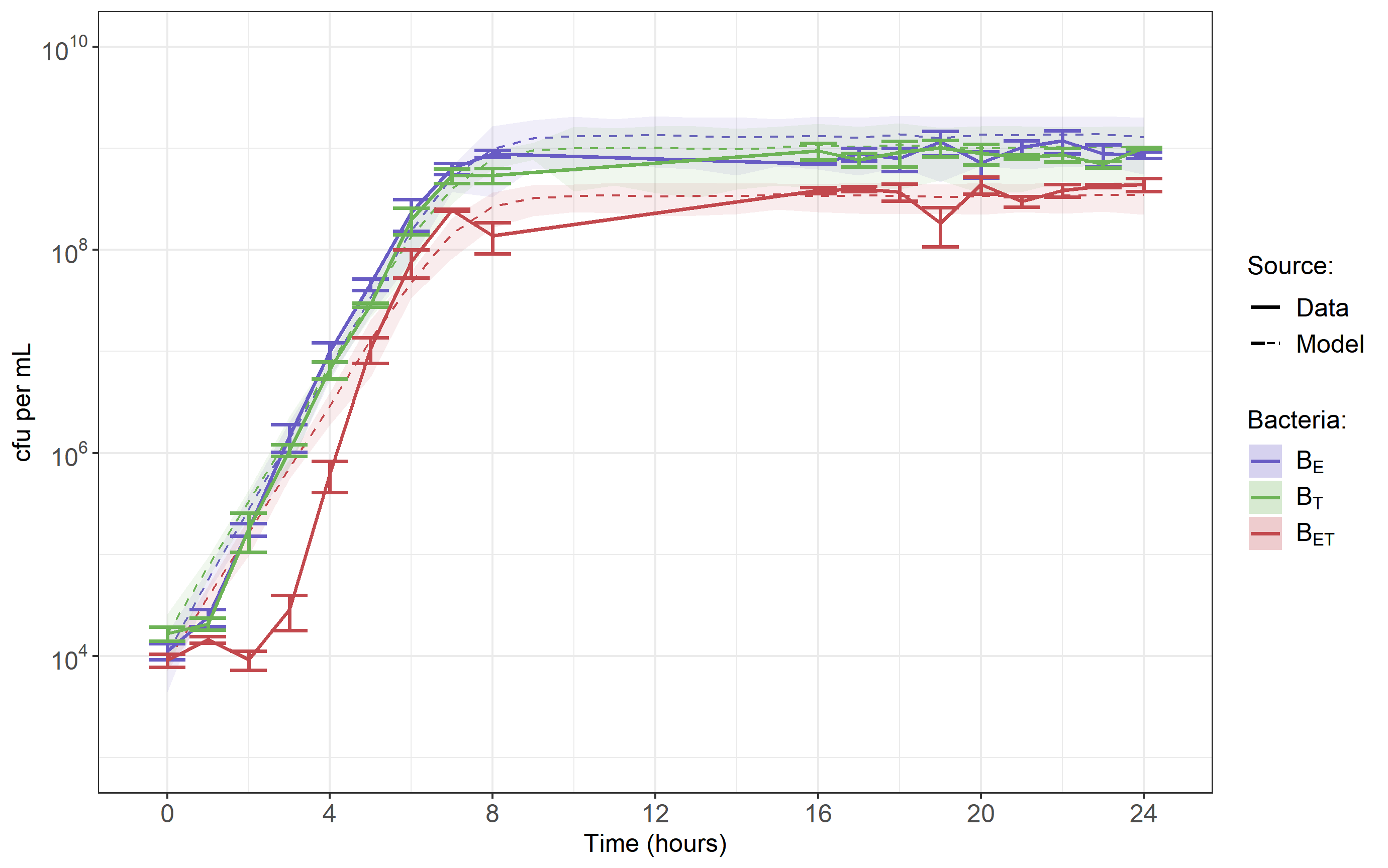

Supplement: FIG S2 [file msystems.00135-22-sf002.tif]

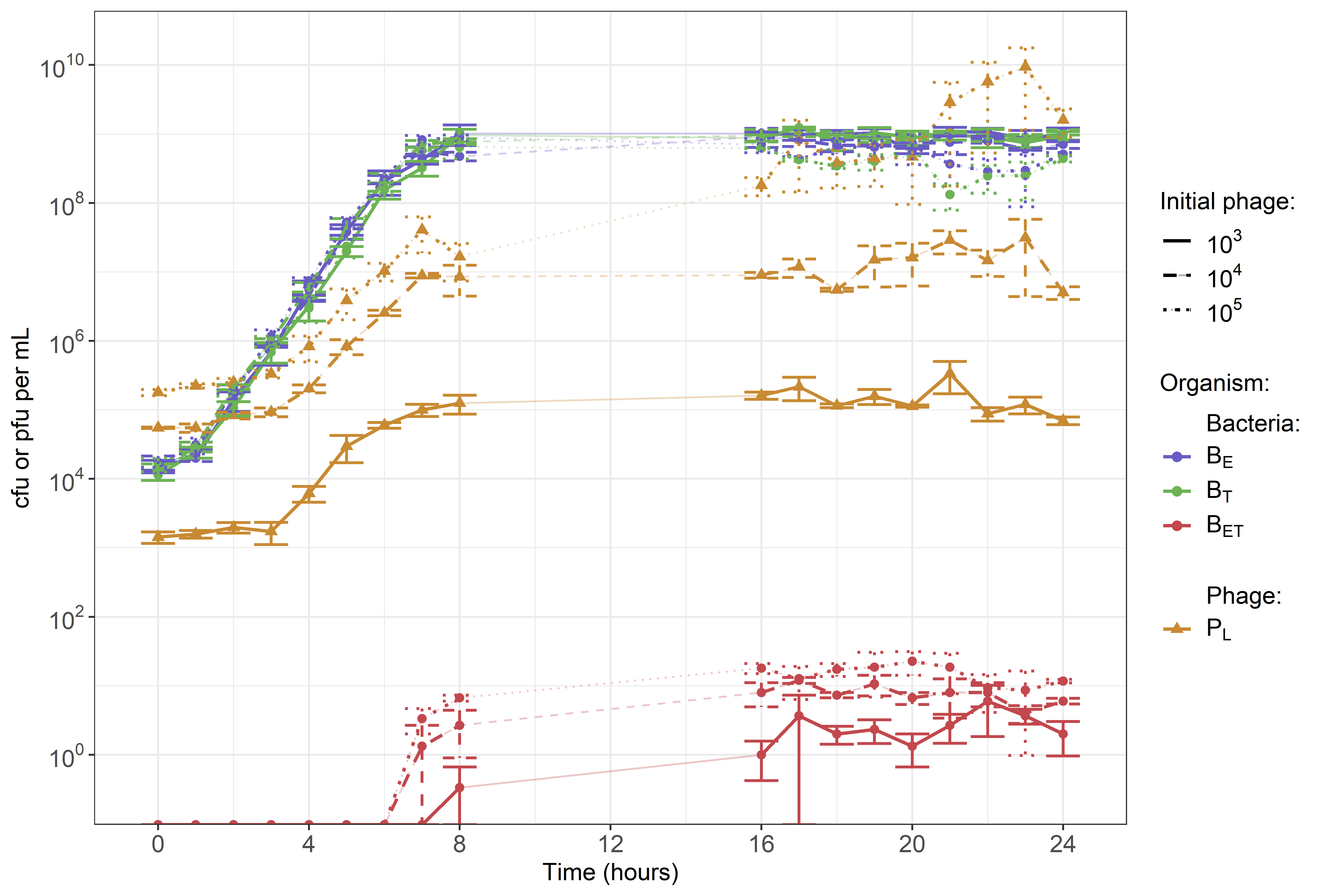

Supplement: FIG S3 [file msystems.00135-22-sf003.tif]

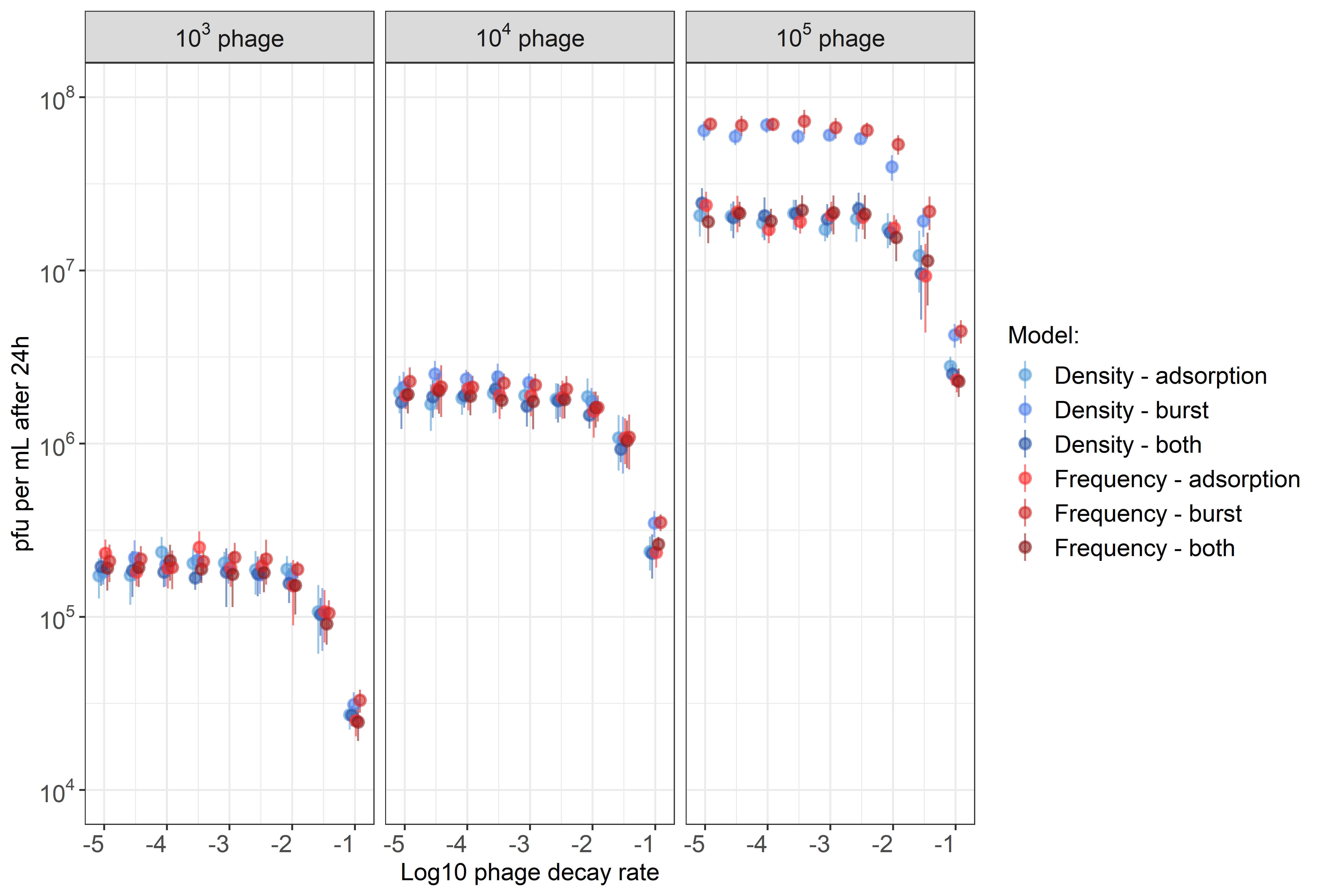

Supplement: FIG S4 [file msystems.00135-22-sf004.tif]

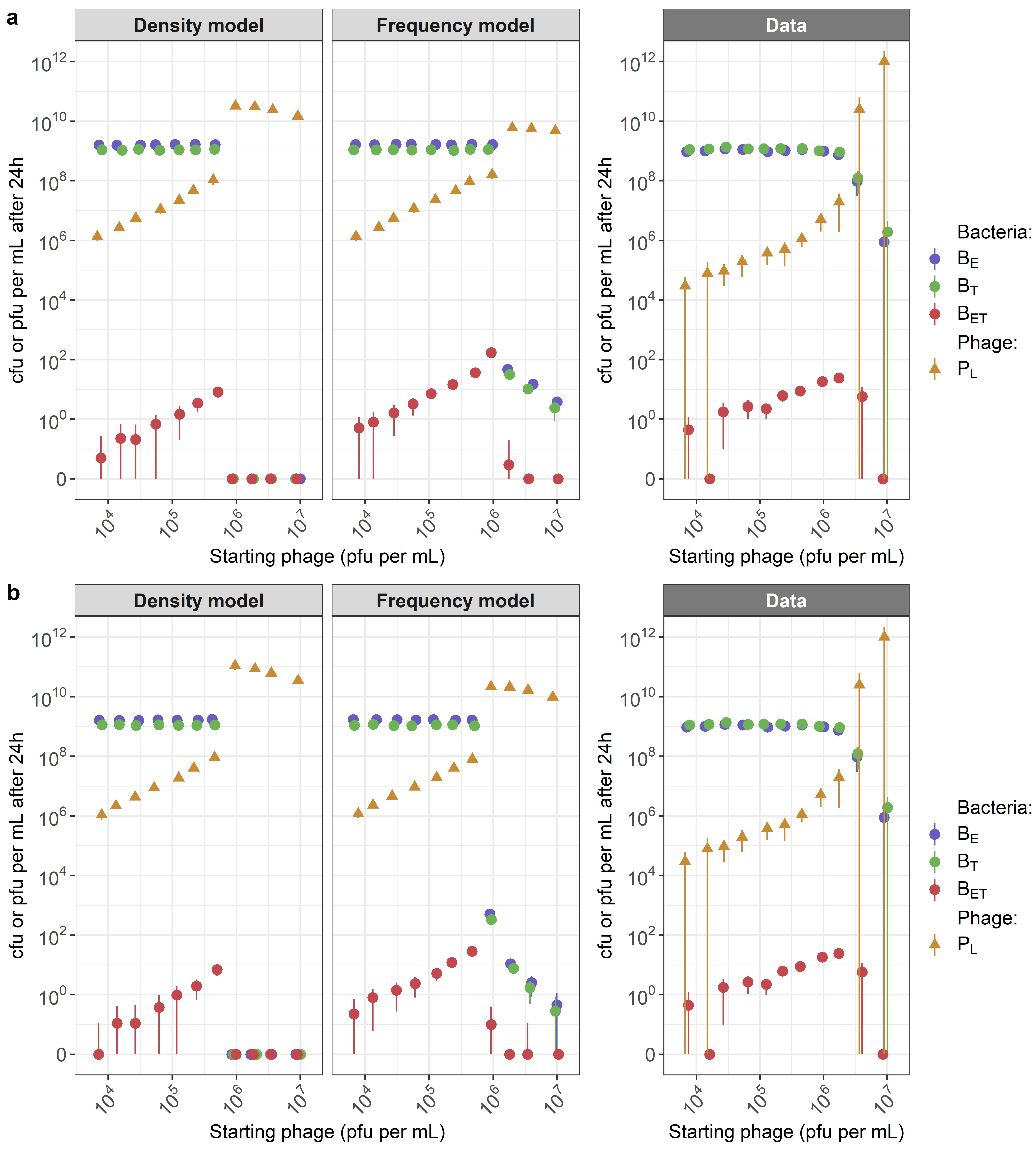

Supplement: FIG S5 [file msystems.00135-22-sf005.tif]

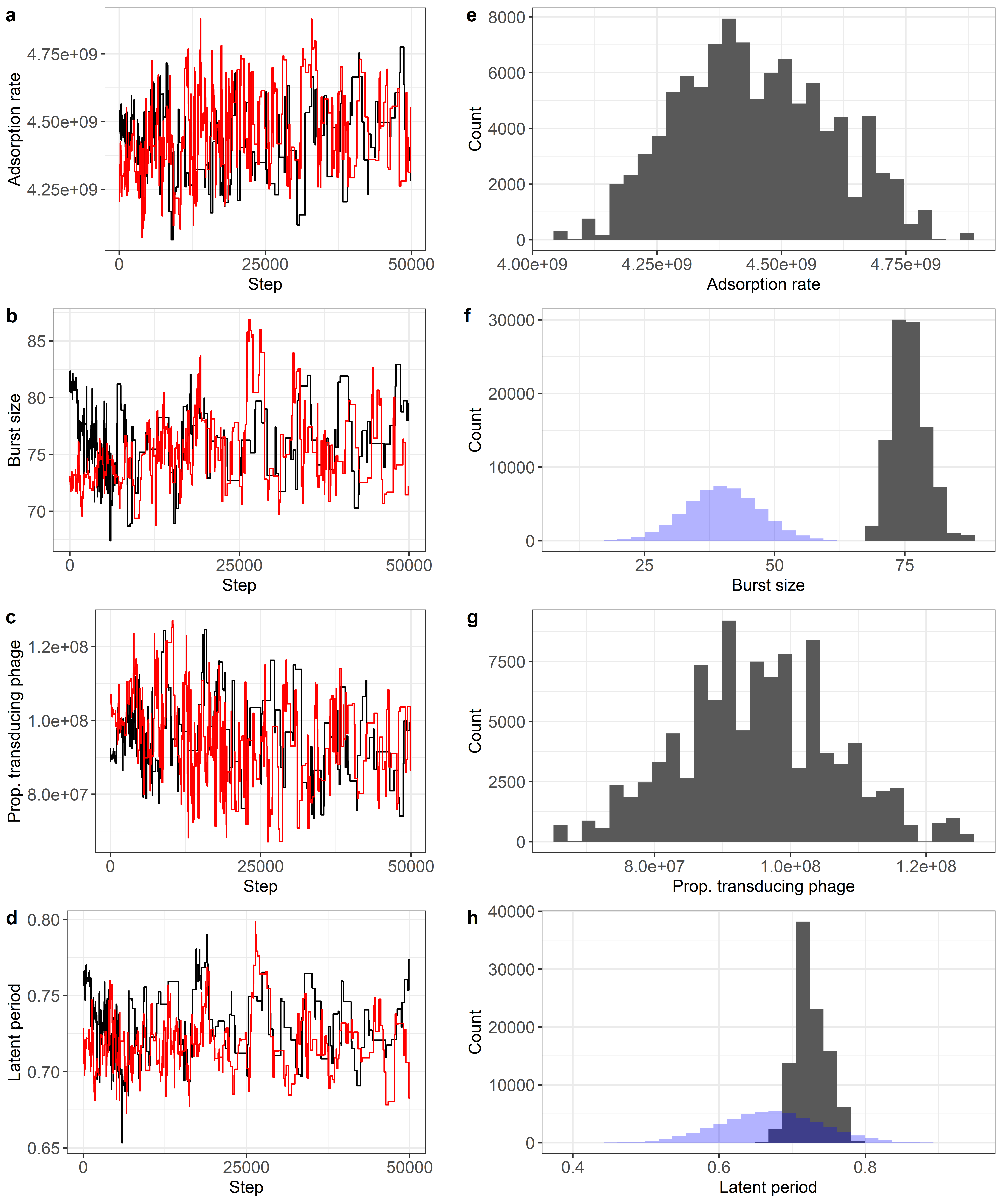

Supplement: FIG S6 [file msystems.00135-22-sf006.tif]
